# Imaging of Osteoarthritic Human Articular Cartilage using Fourier Transform Infrared Microspectroscopy Combined with Multivariate and Univariate Analysis

**DOI:** 10.1038/srep30008

**Published:** 2016-07-21

**Authors:** J. Oinas, L. Rieppo, M. A. J. Finnilä, M. Valkealahti, P. Lehenkari, S. Saarakkala

**Affiliations:** 1 Research Unit of Medical Imaging, Physics and Technology, Faculty of Medicine, University of Oulu, Finland; 2 Medical Research Center, University of Oulu and Oulu University Hospital, Finland; 3 Department of Applied Physics, University of Eastern Finland, Kuopio, Finland; 4 Department of Surgery, Oulu University Hospital, Finland; 5 Research Group of Cancer and Translational Medicine, Faculty of Medicine, University of Oulu, Finland; 6 Department of Diagnostic Radiology, Oulu University Hospital, Finland

## Abstract

The changes in chemical composition of human articular cartilage (AC) caused by osteoarthritis (OA) were investigated using Fourier transform infrared microspectroscopy (FTIR-MS). We demonstrate the sensitivity of FTIR-MS for monitoring compositional changes that occur with OA progression. Twenty-eight AC samples from tibial plateaus were imaged with FTIR-MS. Hyperspectral images of all samples were combined for K-means clustering. Partial least squares regression (PLSR) analysis was used to compare the spectra with the OARSI grade (histopathological grading of OA). Furthermore, the amide I and the carbohydrate regions were used to estimate collagen and proteoglycan contents, respectively. Spectral peak at 1338 cm^−1^ was used to estimate the integrity of the collagen network. The layered structure of AC was revealed using the carbohydrate region for clustering. Statistically significant correlation was observed between the OARSI grade and the collagen integrity in the superficial (*r* = −0.55) and the deep (*r* = −0.41) zones. Furthermore, PLSR models predicted the OARSI grade from the superficial (*r* = 0.94) and the deep (*r* = 0.77) regions of the AC with high accuracy. Obtained results suggest that quantitative and qualitative changes occur in the AC composition during OA progression, and these can be monitored by the use of FTIR-MS.

Articular cartilage (AC) is a specialized connective tissue that covers the ends of long bones. AC redistributes the loads directed on bones and, together with synovial fluid, provides almost frictionless articulation between the bones^1^,^2^. The main constituents of AC are type II collagen, proteoglycans (PGs) and interstitial water^1^. Degradation of the extracellular matrix (ECM) of AC is a hallmark of the initiation of osteoarthritis (OA)^3^. In the early stages of OA, depletion of PGs, especially in the superficial layer, and alterations in the orientation of the collagen fibril network have been reported^4^,^5^,^6^,^7^. Wear and degradation of the entire AC tissue and the possible formation of reparative tissue including fibrocartilage are typical signs of late stage OA^8^,^9^,^10^.

Different non-invasive or minimally invasive imaging methods, such as magnetic resonance imaging (MRI) and computed tomography (CT), have been used clinically to investigate the degenerative stage of AC in OA^11^,^12^,^13^. Typically, only the morphology of AC, *i.e.*, wear of the tissue, can be detected with routine MRI and CT protocols in clinical practice. There is an increased interest in developing new imaging protocols to evaluate compositional changes in AC, which precede morphological changes. For instance, it is possible to detect the depletion of PG and alterations in the collagen network organization with quantitative MRI using T_1ρ_ weighting and T_2_ mapping^14^,^15^. Contrast-enhanced CT can also be used to detect PG content in AC^16^. However, these non-invasive or minimally invasive methods that are capable of evaluating tissue-level changes in AC are still in the developmental stage.

When considering very early OA compositional changes in AC, neither MRI nor CT provides adequate sensitivity. Thus, more direct approaches that are applicable during arthroscopic examination have been introduced for OA diagnostics. These include, *e.g*., optical coherence tomography (OCT), ultrasound (US) imaging^17^ and near infrared (NIR) spectroscopy, all of which are still in the investigational stage and are not in routine clinical use^18^,^19^. Spectroscopic methods are especially promising, as they allow the direct assessment of the composition of hyaline cartilage^20^, thus, providing the possibility to detect early OA changes^21^. However, NIR spectroscopy does not provide similar high-level molecular sensitivity that is achievable using the mid-infrared region^22^. High molecular sensitivity is an essential requirement not only to improve OA diagnostics but also to provide further understanding of the biochemical changes during the development and progression of OA.

Fourier Transform Infrared (FTIR) spectroscopy is a convenient method for investigating the macromolecular composition of biological samples^23^,^24^. FTIR spectroscopy is based on the absorption of infrared light by molecules at characteristic frequencies. FTIR microspectroscopy (FTIR-MS), which enables chemical imaging, is performed by combining a FTIR spectrometer with a conventional light microscope. Consequently, every pixel in an acquired image contains an FTIR absorption spectrum. FTIR images can be analyzed pixel-by-pixel to resolve the spatial distribution and structure of multiple biochemical components, *e.g.*, collagen and PGs in the ECM, from an unstained histological section within a single imaging session^25^,^26^,^27^.

Univariate spectral analysis methods form the basis for analysis of FTIR-MS data^27^. Camacho *et al*.^28^ demonstrated in their pioneering work that collagen and PG contents in AC could be estimated using univariate analysis^28^. Furthermore, the ratio of the integrated areas under CH_2_ side-chains (1300–1360 cm^−1^) and the amide II (1485–1585 cm^−1^) regions was shown to decrease when the collagen network is degraded. The ratio parameter, which is considered a measure of collagen integrity, was later shown to decrease as the OA grade increases^4^,^27^,^29^. It is notable that there are no published studies in which these univariate parameters have been quantitatively correlated with the histopathological OA grade in human samples.

Univariate analysis methods are simple to use, but they discard vast amounts of spectral information. More sophisticated multivariate analysis methods, which derive information from the entire spectrum, could provide more detailed information about the biochemical composition of AC. Partial least squares regression (PLSR) is a multivariate regression method that constructs new variables to explain the co-variance between the reference data and the spectra^30^. PLSR has been used to predict the biomechanical properties^31^ and composition^32^,^33^ of AC from FTIR-MS data, and the status of OA in human and rabbit AC from infrared fiber optic probe data^34^,^35^,^36^. However, unlike FTIR-MS, infrared fiber optic probe measurements are limited to the superficial cartilage. To the best of our knowledge, PLSR has not been used to predict the histopathological state (*i.e*., OARSI grade^10^) of human AC tissue from FTIR-MS data.

Cluster analysis belongs to another class of multivariate analysis methods. Clustering algorithms classify spectra into groups by minimizing differences within groups and maximizing differences between groups. This classification can be used for segmentation of FTIR images^37^,^38^,^39^,^40^,^41^,^42^,^43^. It is well known that AC has a layered structure and it is typically divided into three zones from the articulating surface to subchondral bone based on the orientation of collagen fibrils and the biochemical composition of the ECM^2^,^44^,^45^. Earlier, clustering algorithms have been used to reveal zonal structure in healthy bovine and rabbit AC^40^. Furthermore, cluster analysis has also been used to differentiate intact and repaired AC from patellar grooves in rabbit femurs^41^. However, cluster analysis has not yet been used to reveal zonal structure in human AC.

The first aim of the present study was to investigate the quantitative and qualitative changes in the FTIR spectra of human AC as a function of the histological OA grade (OARSI grading). We hypothesized that the zonal structure of the AC and changes in the macromolecular structures during OA progression could be detected when cluster analysis is conducted simultaneously on the spectra of all samples. We expected that particular clusters would concentrate on samples with a certain OARSI grade. The second aim of the study was to construct multivariate PLSR models to predict the histopathological OARSI grade of the human AC tissue. We hypothesized that a more accurate prediction would be obtained using the spectral information from the surface layer than from the deep layer of the AC. In addition, we hypothesized, as suggested by earlier literature, that negative correlations between the OARSI grade and every univariate parameter (amide I, carbohydrate region, collagen integrity and second derivative peaks 1202 cm^−1^ and 1064 cm^−1^) would be observed^4^,^21^,^29^,^34^.

## Results

### Cluster analysis

**Figure 1:**
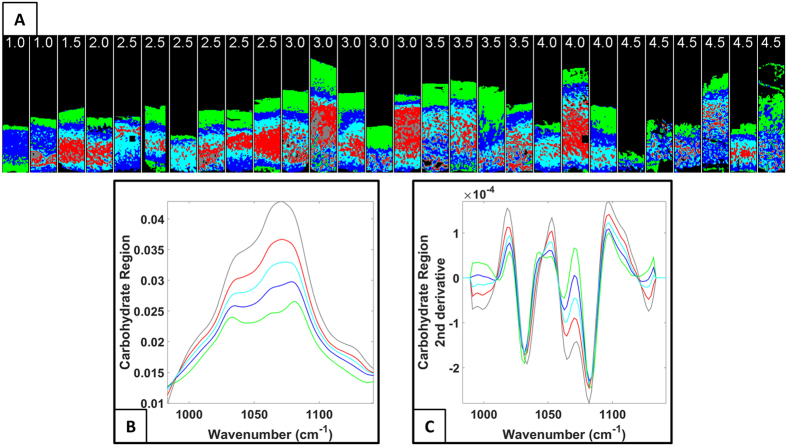
(**A**) Cluster images obtained using the carbohydrate region for clustering. The characteristic layered structure of AC can be observed in the cluster images. The OARSI grade is indicated above each sample. (**B**) Average spectra of each cluster. (**C**) The second derivative spectra of each cluster. Major differences between the second derivative spectra can be seen around the peak at 1060 cm^−1^. The colors of the spectra correspond to the cluster colors.

**Figure 2:**
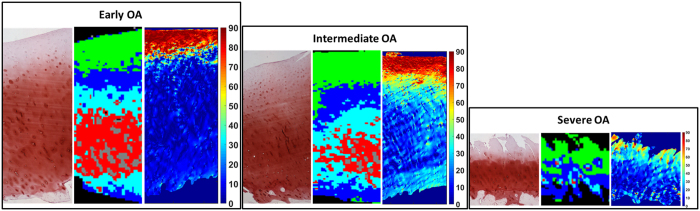
Safranin O, cluster and PLM images from three samples with different severity of OA (Early OA = OARSI grade 1.5, Intermediate OA = OARSI grade 3.5 and Severe OA = OARSI grade 4.5). The corresponding cluster images from the carbohydrate region are extracted from the combined hyperspectral data. Black pixels in the cluster image are due to the removal of poor quality spectra.

After careful preliminary analyses of the large data matrix (including all the samples), five clusters were found to be optimal for revealing the layered structure of AC. Different spectral regions were evaluated by clustering to expose the layered structure of AC tissue and to explore the changes in the resulting cluster images during OA progression. The layered structure of AC in each sample was observed in the cluster images using the carbohydrate region for clustering (Fig. 1A). The lowest intensity was observed in the average raw spectra of the green cluster in the carbohydrate region, which indicates the lowest relative PG content (Fig. 1B). Major spectral differences between the clusters were observed in the spectral region from 1058 cm^−1^ to 1080 cm^−1^ in the second derivative spectra when the carbohydrate region was used for cluster analysis (Fig. 1C). In Fig. 2, Safranin O-stained sections, cluster images that were obtained using the carbohydrate region and PLM images of three representative samples with different OA grades are presented (Fig. 2). The corresponding images for all 28 samples can be found in the Supplementary Material. Similar features can be observed in the images. For example, the superficial, the middle and the deep layers of the AC are visible in PLM and cluster images. Furthermore, the green cluster in the cluster images complies with the Safranin-O image. On the surface of the Safranin-O images, the amount of staining is low. Correspondingly, the green cluster in the cluster images is located in the superficial layer of the AC (Fig. 2). A visual correlation between the OARSI grade and the cluster image obtained from the carbohydrate region was not observed. Similarly, when using the extended carbohydrate region, the combination of the amide I and amide II regions, or the combination of the amide I, amide II and carbohydrate regions, there was no clear correspondence between the cluster images and the OARSI grade.

### Partial least squares regression

**Figure 3:**
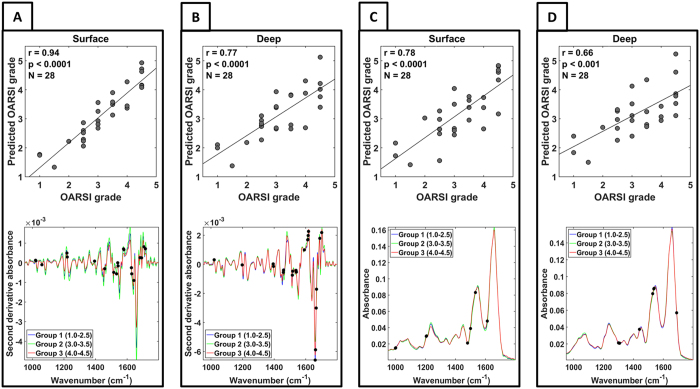
Scatter plots between the reference values and the predicted values of the OARSI grade and the average spectra of each pooled OA group. The black dots in the spectra indicate the wavenumbers that were selected to the PLSR models by the CARS algorithm. (**A,B**) the plots from the models that used second derivative spectra. (**C,D**) the plots from the model that used raw vector normalized spectra.

PLSR predicted the OARSI grade more accurately from the second derivative spectra (Fig. 3A,B) (*r*_*Surface*_ = 0.94, *p* < 0.0001, *r*_*Deep*_ = 0.77, *p* < 0.001) than from the raw spectra (Fig. 3C,D) (*r*_*Surface*_ = 0.78, *p* < 0.0001, *r*_*Deep*_ = 0.66, *p* < 0.001) in both the surface and the deep layers of AC (Fig. 3). The percent errors for the second derivative spectrum models were also lower (*E*_*surface*_ = 11.51%, *E*_*deep*_ = 17.66%) than for the raw spectrum models (*E*_*surface*_ = 18.30%, *E*_*deep*_ = 21.25%). Overall, the OARSI grade was more accurately predicted from the surface layer than from the deep layer of AC. The number of latent variables (LVs) varied from two to six between the models. With the spectra from the surface, six and five LVs were used in the models built from the second derivative and the raw spectra, respectively. Correspondingly, five and two LVs were used in the models in the deep layer. The CARS algorithm selected eight optimal variables (wavenumbers) for the PLSR models in both the surface and the deep layer when the raw spectra were used. When the second derivative spectra were used, 21 and 20 variables were selected to the models for the superficial and the deep layer, respectively. The variables are marked with black dots in the spectra of corresponding model (Fig. 3A–D).

### Univariate analysis

**Figure 4:**
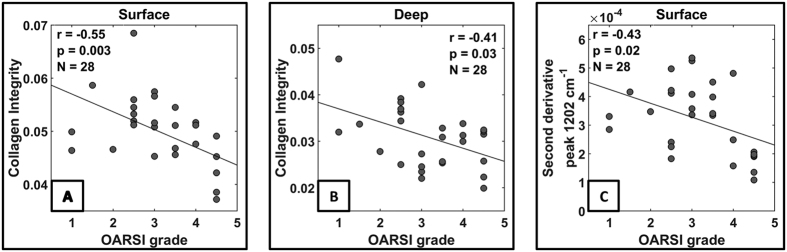
Statistically significant negative correlations were observed between the OARSI grade and the collagen integrity parameter in both the surface (**A**) and the deep layer (**B**), and the second derivative peak 1202 cm^−1^ in the superficial layer (**C**).

Statistically significant correlations were not observed between the OARSI grade and the amide I region, the carbohydrate region, the carbohydrate region normalized with the amide I region or the second derivative peak 1064 cm^−1^ (PG) in either of the analyzed zones (surface and deep). However, statistically significant negative correlations were observed between the OARSI grade and the collagen integrity parameter in both the surface and the deep zones (surface: *r* = −0.55, *p* < 0.003, deep: *r* = –0.41, *p* = 0.03) (Fig. 4A,B). A statistically significant negative correlation (*r* = *−*0.43*, p* = 0.02) was also found between the OARSI grade and the second derivative peak at 1202 cm^−1^ (collagen) in the surface layer of AC (Fig. 4C).

**Figure 5:**
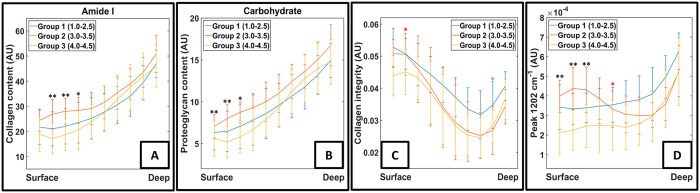
For the group comparison, the samples were pooled in three groups. Group 1 (Early OA) include the grades from 1.0 to 2.5 (*n* = 10), group 2 (Intermediate OA) include the grades from 3.0 to 3.5 (*n* = 9), and the group 3 (Severe OA) include the grades from 4.0 to 4.5 (*n* = 9). (**A**) Collagen content estimated by the amide I region of the FTIR spectra. (**B**) PG content estimated by the carbohydrate region of the FTIR spectra. (**C**) Collagen integrity as a function of tissue depth. (**D**) Second derivative peak 1202 cm^−1^ as function of tissue depth. In the plots, black stars indicate the difference between the groups 2 and 3, red stars indicate the difference between the groups 1 and 3, and green stars indicate the difference between groups 1 and 2. *p < 0.05, **p < 0.01.

Statistically significant differences were found between the OA groups (group 1 = early OA, group 2 = intermediate OA and group 3 = severe OA) in all univariate parameters (amide I region, carbohydrate region, collagen integrity and the second derivative peak 1202 cm^−1^) (Fig. 5). In the amide I and carbohydrate regions, statistically significant differences were observed between intermediate OA and severe OA in the surface and middle layers (Fig. 5A,B). Collagen integrity was significantly different between early OA and severe OA in the surface layer and between early OA and intermediate OA in the deep layer (Fig. 5C). The peak at 1202 cm^−1^ exhibited significant differences between intermediate OA and severe OA in the surface layer and between early OA and severe OA in the middle layer (Fig. 5D).

## Discussion

In the present study, human AC samples with various histological grades of OA were studied using FTIR-MS. The aims of the study were to detect quantitative and qualitative osteoarthritic changes in the FTIR spectra of AC. We hypothesized that the layered structure of AC could be observed from the cluster analysis images and that the particular cluster will concentrate on a certain OARSI grade. The results obtained indicate that the structural and compositional changes caused by OA can be observed with both the uni- and multivariate analyses of spectral data. The present study is the first to conduct cluster analysis on FTIR images of multiple human AC samples simultaneously, *i.e.*, by combining hyperspectral data from several individual samples into one large data matrix. This is advantageous because it allows direct comparison of the clusters between the samples. Unfortunately, compared to our original hypothesis, cluster analysis did not reveal any OA-related clusters. However, the PLSR models demonstrated that OA-related changes occur in both the surface and the deep layers of AC.

The layered structure of AC in cluster images is similar to the structure observed in PLM orientation images. Based on the visual evaluation of cluster images from all the spectral regions, the best correspondence with PLM images was observed when the carbohydrate region was used. It can be assumed that the relative amounts of collagen and PGs are the main differences between the clusters in the carbohydrate region. For example, the green cluster is found in the superficial layer of AC in the cluster images (Fig. 1A). The AC surface contains low amounts of PGs. Furthermore, the mean spectra of clusters (Fig. 1B) demonstrate that the overall absorbance in the carbohydrate region is at its lowest in the green cluster. Therefore, it can be assumed that the green cluster indicated the regions with the lowest relative amounts of PGs in the sample sections. In contrast, by examining the overall absorbance of the mean spectra in the carbohydrate region (Fig. 1B), the relative PG content is at its highest in the grey cluster that is found in the deep zone. The other three clusters lie between the grey and green clusters in terms of their relative PG content (Fig. 1B). In the second derivative spectra, the main differences are observed in the spectral region around the peak at 1060 cm^−1^ (Fig. 1C). The vibrations from this region have been shown to arise from the C–O stretching vibrations of carbohydrate residues in collagens and in PGs and from 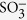 stretching vibration in sulfated glycosaminoglycans^46^,^47^,^48^. Because Safranin-O is a cationic dye, it binds to the negatively charged PGs, and therefore, it can be used for observing the distribution of PGs^49^. When the cluster image is compared with the Safranin-O stained sections, the green cluster can be observed to concentrate in the regions where the amount of the Safranin-O stain is low. This supports the idea that the clustering result follows the relative amount of the PGs.

Because the layered structure of AC is traditionally defined by the organization of the collagen network, the amide region was presumed to reveal the layered structure more clearly than the other spectral regions that were investigated. The characteristic layered structure of healthy rabbit and bovine AC could be revealed with clustering algorithms using the amide I, amide II or carbohydrate region^40^. In earlier studies, the clustering results followed the concentration of AC constituents and the organization of the collagen network. In our study with human AC, the layered structure was not evident in all samples when using the amide I or amide II regions alone. One factor that affects the amide I and amide II regions is the traces of water vapor in the measurement chamber. Water vapor displays several sharp bands that overlap the amide I and amide II regions^51^. A second factor that may explain the difference is the different species investigated (rabbit vs. human). Finally, in the study by Kobrina *et al*.^40^, the clustering was performed on one sample at a time, whereas in this study clustering was performed simultaneously on all samples.

Compared to our original hypothesis, cluster analysis did not detect a cluster that is directly related to osteoarthritic changes in AC. K-means clustering is an unsupervised algorithm, *i.e.*, it was not specifically taught to find osteoarthritic changes, but it groups the spectra according to their similarities based on Euclidean distance. The clusters that are detected seem to be related to variations in the relative PG and collagen contents in the AC. It is possible that clusters associated with OA would be found if the number of clusters would be significantly increased. However, the interpretation of all clusters would become more difficult as their number increases; therefore, based on our preliminary analyses it was not seen feasible in this study.

Although our clustering results did not correlate strongly with the OARSI grade, the PLSR analysis demonstrated that the OARSI grade can be predicted from the FTIR spectra. It is often suggested that the first signs of OA in AC are observed in the superficial layer^5^,^6^. A competing view suggests that OA could be initiated in the subchondral bone, as during OA changes also occur in the subchondral bone^52^. In our study, the OARSI grade was predicted more accurately from the superficial layer than the deep layer (as indicated by both the correlation and the percentage error analyses). This supports the idea that the most significant biochemical changes occurred in the surface of the AC in this relatively heterogeneous sample set. The OARSI grading system does not directly take into account the biochemical changes that occur in AC. However, as the PLSR satisfactorily predicted the OARSI grade from the deep AC layer, biochemical changes may also occur in the deep layers of AC during OA progression. Furthermore, as expected, the results of the second derivative spectra, which enhance the separation of the overlapping peaks^53^, resulted in a more accurate prediction of the OARSI grade.

With regard to univariate analysis of the FTIR spectra, no significant correlations were observed between the OARSI grade and the integrated absorbances of the amide I and carbohydrate regions, even though an earlier study suggested that quantitative correlations may exist^54^. However, a significant correlation was observed in the superficial layer between the OARSI grade and the second derivative peak at 1202 cm^−1^, which can be used to monitor the collagen content^53^. The difference in the results between the amide I and the second derivative peak might be due to the better separation of overlapping peaks obtained using second derivative spectroscopy^53^. However, the lack of a correlation between the collagen content and the OARSI grade would not be that surprising, as some earlier studies did not detect a difference in the collagen content between healthy and OA human AC^55^. This suggests that the biochemical changes in AC are not linearly related to the OARSI grade. Group comparisons revealed that changes are observed primarily in the first 40% of the thickness from the cartilage surface: the collagen content, indicated by both the amide I region and the second derivative peak at 1202 cm^−1^, and the PG content are significantly lower in severe OA compared to intermediate OA. Surprisingly, the collagen and PG contents appear lower in early OA compared to intermediate OA, although the differences were not statistically significant. However, if this finding were true, it may be explained by the increased levels of collagen and PG expression in chondrocytes after initial cartilage damage^56^,^57^.

Our results clearly indicated that the collagen integrity parameter decreases while the OARSI grade increases in both the superficial and deep zones of AC. This finding is in agreement with an earlier study, which demonstrated a statistically significant difference in the collagen integrity parameter between the Collins grade 1 (nearly normal) and grade 3 (degraded) human AC samples^29^. The collagen integrity parameter is believed to be sensitive to the triple helical structure of type II collagen. Therefore, it can be used to assess the degradation of collagen^29^,^58^. The enzyme collagenase degrades the collagen network during the development of OA, especially near the focal lesions^56^,^57^. The collagen integrity parameter has been validated earlier specifically with collagenase treated samples^59^. Therefore, the collagen integrity parameter, derived from the FTIR spectral data, may provide a measure of the denaturation level of the collagen in AC. In earlier studies, only group comparisons between the collagen integrity and OA stage have been reported^4^,^29^,^59^. In this study, a correlation between the collagen integrity and the OARSI grade was observed, which supports the sensitivity of this univariate parameter for OA changes. The collagen integrity parameter is at a similar level in early and intermediate OA in the superficial layer. However, the collagen integrity in the intermediate OA group decreases quickly to the same level with the severe OA group in the deeper layers of AC. The differences in the collagen integrity between the groups were mostly statistically insignificant probably due to the low number of samples that were evaluated.

In the OARSI system, the condition of the AC surface mainly determines the OA grade of the sample. The surface layer of AC is already completely degraded in the OARSI grade of 4.0^10^. This may partially explain the lack of correlation between the OARSI grade and the amide I or carbohydrate regions in the superficial layer. Furthermore, the OARSI system does not directly take into account the changes in the collagen or PG content in AC during OA progression. Therefore, significant correlations may not be expected especially in a small sample set. In addition, our sample set consisted of samples with OARSI grades higher than 1.0, which is the threshold for OA in the OARSI grading system^10^. Therefore, we did not have completely healthy specimens in our sample set, and we could not study the very early OA changes in the AC composition. It is possible that most systematic changes in the biochemical content occur during the early stages of OA. Another limitation of the study is the relatively low number of samples (*N* = 28) that were evaluated. Further studies with a larger sample size are required, as multivariate methods benefit greatly from a larger sample population.

## Materials and Methods

### Samples

The ethical Committee of the Northern Ostrobothnia Hospital District, Oulu, Finland (191/2000 and 14–291) approved all experimental procedures, and the study was performed in accordance with the approved guidelines. Human AC samples (*n* = 28) were collected from patients (*N* = 26) undergoing total knee arthroplasty at Oulu University Hospital. Informed consent was obtained from all patients. Cylindrical osteochondral blocks were drilled from human tibial plateaus with different visual grades of OA. Subsequently, the blocks were fixed in formalin and decalcified in ethylenediaminetetraacetic acid (EDTA). After decalcification, the blocks were dehydrated and embedded in paraffin. For the FTIR-MS measurements, 5 μm thick sections were cut using a microtome (Thermo Scientific, Microm HM 355S, Waltham, MA, USA) and, after removal of paraffin with xylene, placed on 0.5 mm thick Zinc-Selenide (ZnSe) windows (Crystran Ltd., Poole, United Kingdom). In addition, three adjacent sections were stained with Safranin-O and evaluated according to the OARSI histopathology grading system^10^. Briefly, the OARSI system divides the OA into seven main grades (0–6), where healthy cartilage is graded as 0, and the cartilage is denuded at grade 5. The highest grade 6 includes bone remodeling. In addition, subgrades of 0.5 were also used in the grading. Samples with an OARSI grade greater than 4 were not used in this study because the AC is already completely worn off at grade 5.

### Fourier Transform Infrared Microspectroscopy (FTIR-MS)

FTIR-MS measurements were conducted using an FTIR spectrometer (Tensor 27, Bruker Inc., Billerica, MA, USA) coupled with a Bruker Hyperion 3000 microscope (Bruker Inc., Billerica, MA, USA) equipped with a focal plane array (FPA) 64 × 64 detector. The system was controlled with the manufacturer’s Opus 7.2 software. First, panoramic images were collected in visual imaging mode using the same microscope. From the visual images, 500 μm wide areas from the cartilage surface to subchondral bone (500–2000 μm) were selected for FTIR-MS. All measurements were conducted in transmission mode, where incident infrared light is beamed through the sample section, and an FTIR absorption spectrum is obtained for every pixel. The spectral region from 900 cm^−1^ to 3800 cm^−1^ (corresponding to wavelengths from 2.6 μm to 11.1 μm) was collected. The spectral resolution was set to 4 cm^−1^, and each spectrum was averaged 16 times. Spatial resolution was set to 21.6 μm using pixel binning. The sample chamber was purged with dry air to stabilize measurement conditions.

### Data preprocessing

All preprocessing and analysis of spectral data were performed with custom-made MATLAB (MathWorks Inc., MA, USA) scripts and the commercial MATLAB toolbox Cytospec 2.00.01 (Berlin, Germany). A Resonant Mie Scattering Correction (RMieSC) algorithm was used to remove the scattering effects from the spectra^60^. Predefined thresholds for peak intensity and signal-to-noise ratios (SNR) were used as quality criteria, and poor quality spectra were removed from the dataset using the quality test functions in Cytospec. Principal component analysis (PCA) was used to detect the cartilage-bone interface. PCA is a method that creates a new set of linearly uncorrelated variables named principal components (PCs)^61^. PCs are classified so that the first PC explains most of the variance in the data and the second PC the second most and so on. PCA was performed using the carbohydrate region of the data matrix that included all the samples. A gray scale image for discriminating the bone from the AC was created using score values of the first PC. The score image was visually inspected and the bone spectra were manually removed from the dataset. After the removal of bone spectra, the spectra were truncated to a fingerprint region (950–1800 cm^−1^) that contains detailed information about proteins and carbohydrates. When calculating the collagen integrity parameter, a linear two-point baseline correction was performed on the CH_2_ side-chain and the amide II regions individually. Furthermore, for the cluster analysis, the spectra were vector normalized after RMieSC. Thus, the differences in the amounts of material, *i.e.*, the total absorbance, are removed, and clustering is based only on qualitative properties, *i.e.*, the shape of the sample’s spectra. The second derivatives spectra for the univariate and PLSR analyses were calculated using the Savitzky-Golay algorithm with nine smoothing points^62^.

### Cluster analysis

Cluster analysis methods are unsupervised, *i.e.*, no *a priori* information about class memberships is given. The K-means clustering (KMC) algorithm was used in this study. The basic mathematical principle of the KMC algorithm has been presented by MacQueen^63^. The KMC algorithm is a “coarse” method for classifying the spectra because a single spectrum can be a member of only one class (cluster). The algorithm classifies the spectra so that the spectral differences within clusters are minimized and the spectral differences between clusters are maximized. The Euclidean distance is used to define the differences between the spectra^38^. First, the algorithm selects a predefined number (K) of random spectra from the data set as the centroid spectra. Then, the algorithm calculates the distance between every spectrum to each centroid spectrum and classifies the spectra in K classes according to the distances. New centroid spectra are created by calculating the average spectrum for each class. Again, the distances between each spectrum to each centroid spectrum are calculated and new K classes are created. In this study, the number of these iterations was set to 20, which was visually observed to be an adequate number (no notable differences were observed in the resulting cluster images when the number of iterations was increased). As a result, the algorithm generates an image in which one color represents the particular class (cluster)^38^,^39^. Black pixels in the cluster images are due to the removal of poor quality spectra.

The 28 individual hyperspectral data images that were measured were combined into one large data matrix for cluster analysis. Different spectral regions can be used in KMC classifications. In this study, the following spectral regions were evaluated for cluster analysis: the carbohydrate region (985–1140 cm^−1^), the extended carbohydrate region (950–1400 cm^−1^), the amide I region (1585–1720 cm^−1^), the amide II region (1485–1585 cm^−1^), and the combination of the carbohydrate, amide I and amide II regions. Spectral differences between the clusters were investigated by observing the average spectra of each cluster. In addition, second derivative spectra of the cluster averages were also calculated.

First, the zonal structure of AC in the clustering images that were obtained was visually compared with the collagen fibril orientation images obtained using polarized light microscopy (PLM). Second, the obtained cluster images were compared with the Safranin-O stained sections to obtain information about the PG contents of different clusters.

### Partial least squares regression (PLSR)

PLSR is a powerful multivariate analysis method, which is widely used in chemometric analysis. The spectral peaks from different molecular vibrations tend to overlap in FTIR spectra of biological tissues. PLSR is insensitive to such collinear variables and tolerates a large number of variables. PLSR projects the spectral data onto orthogonal latent variables (LVs)^30^. In the present study, the PLSR was used to predict the OARSI grade of each sample using the FTIR spectra as predictors. The models were created separately for the spectral data from the surface layer (0–15% of the thickness from the surface towards the tidemark) and the deep layer (65–100% of the thickness from the surface toward the tidemark). Using the second derivative and the raw spectra, four models were created. Additionally, the competitive adaptive reweighted sampling (CARS) algorithm^64^ was used to select an optimal combination of variables (wavenumbers) for the PLSR model. The maximum number of LVs was limited to 20. Leave-one-out cross-validation was used for validation of the multivariate models. Spearman’s correlation was used to measure the agreement between the reference values and the predicted OARSI grades. In addition, the mean percent error was calculated for each model.

### Univariate analysis

The integrated areas under the amide I and carbohydrate regions as well as the ratio of the integrated areas under the region around the 1338 cm^−1^ peak and the amide II region were calculated for each spectrum for univariate analysis. Additionally, to avoid sample thickness variations, the integrated area of the carbohydrate region was also normalized by the integrated area of the amide I region. Consequently, depth-wise average profiles were calculated for every parameter. The regions of interest for the superficial and the deep layers were selected similarly to those used in the PLSR analysis. From the second derivative spectra, the peaks at 1064 cm^−1^ and 1202 cm^−1^ were used to analyze PG and collagen contents, respectively^53^. The results were compared with the OARSI grade using correlation analysis.

### Polarized light microscopy (PLM)

PLM was used as a reference method when studying the layered structure of AC^65^. All 28 samples were imaged using an Abrio PLM system (CRi, Inc., Woburn, MA, USA) mounted on a conventional light microscope (Nikon Diaphot TMD, Nikon, Inc., Shinagawa, Tokyo, Japan). The Abrio system consists of a green bandpass filter, a circular polarizer, and a computer-controlled analyzer composed of two liquid crystal polarizers and a CCD camera. In a PLM orientation image, each pixel consists of information about the average orientation of the collagen fibrils.

### Statistical analysis

The Spearman’s correlation test was used for all correlation analyses. For the group comparisons, the OARSI grades were pooled into three groups. Group 1 (early OA) included the grades from 1.0 to 2.5 (*n* = 10), group 2 (intermediate OA) included the grades from 3.0 to 3.5 (*n* = 9), and group 3 (severe OA) included the grades from 4.0 to 4.5 (*n* = 9). The Kruskall-Wallis test with pairwise comparison was used to analyze the statistical significance between the groups. The group comparisons were performed for the amide I region, carbohydrate region, collagen integrity and second derivative peak at 1202 cm^−1^. The statistical analyses for the univariate parameters were performed using SPSS 22 software (IBM SPSS Statistics, SPSS Inc., Chicago, IL, USA).

## Additional Information

**How to cite this article**: Oinas, J. *et al*. Imaging of Osteoarthritic Human Articular Cartilage using Fourier Transform Infrared Microspectroscopy Combined with Multivariate and Univariate Analysis. *Sci. Rep.* **6**, 30008; doi: 10.1038/srep30008 (2016).

## Supplementary Material

Supplementary Information
