# Supplementary material for: Imaging of Osteoarthritic Human Articular Cartilage using Fourier Transform Infrared Microspectroscopy Combined with Multivariate and Univariate Analysis
Source: Sci Rep. 2016 Jul 21;6:30008. doi: 10.1038/srep30008 (PMC4956759; doi:10.1038/srep30008)

Imaging of Osteoarthritic Human Articular Cartilage using Fourier Transform Infrared Microspectroscopy Combined with Multivariate and Univariate Analysis

Oinas J., Rieppo L., Finnilä M. A. J., Valkealahti M., Lehenkari P., Saarakkala S.

1. (OARSI grade 1.0)

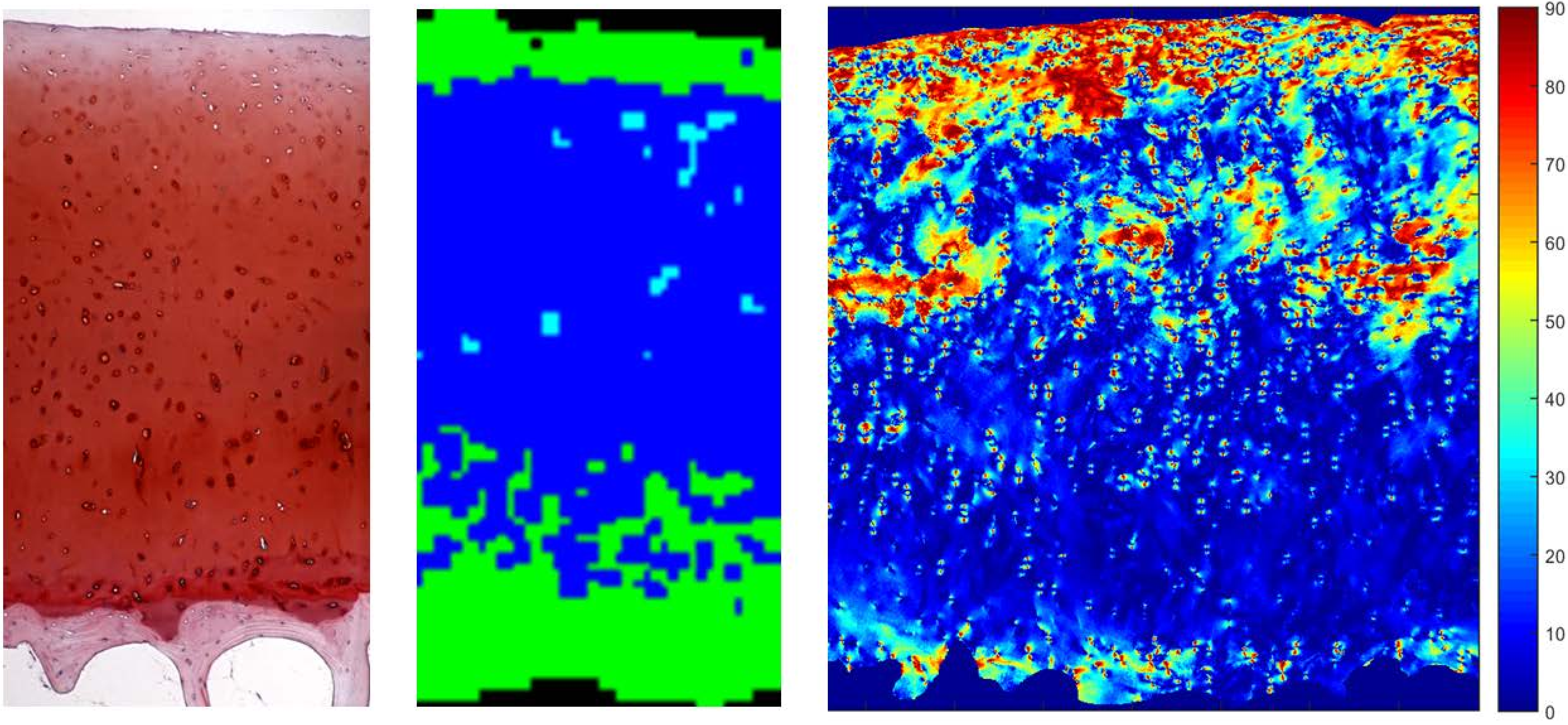

2. (OARSI grade 1.0)

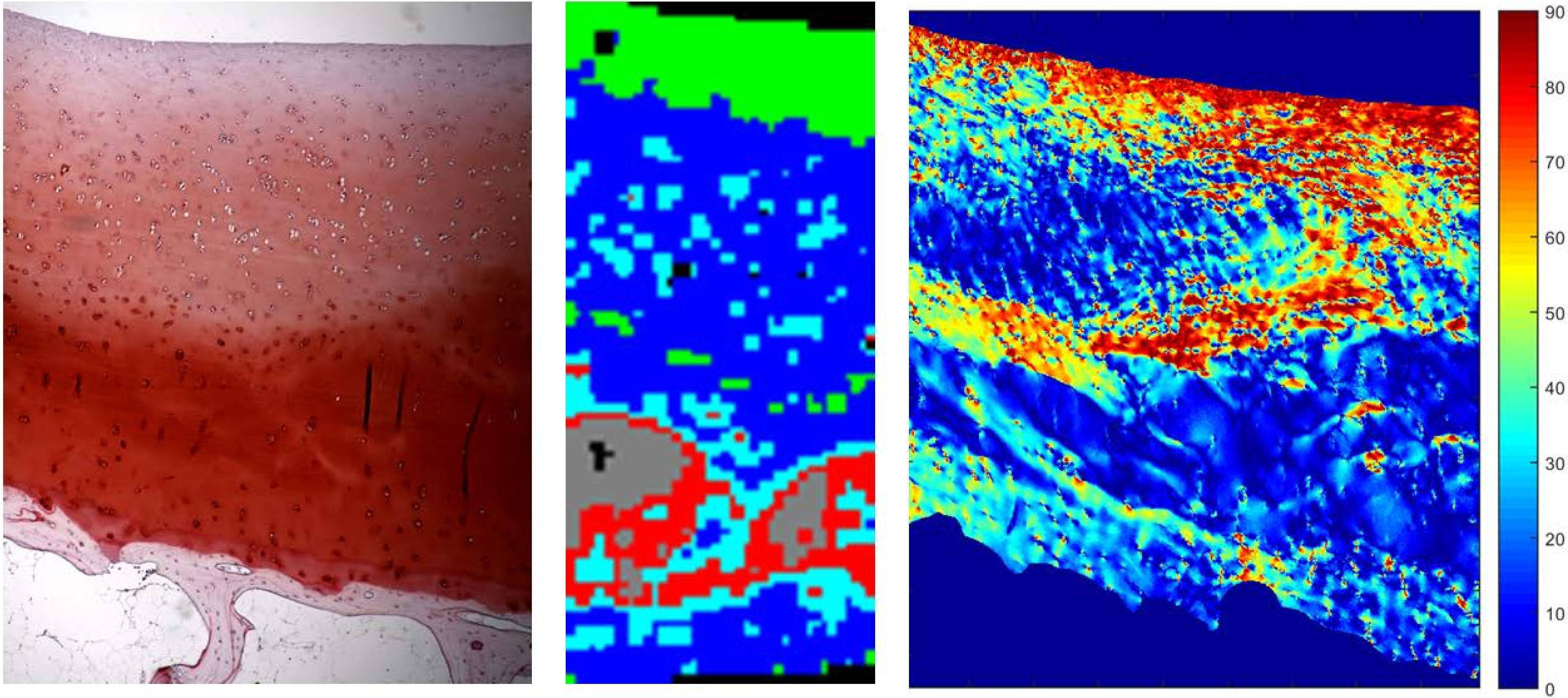

3. (OARSI grade 1.5)

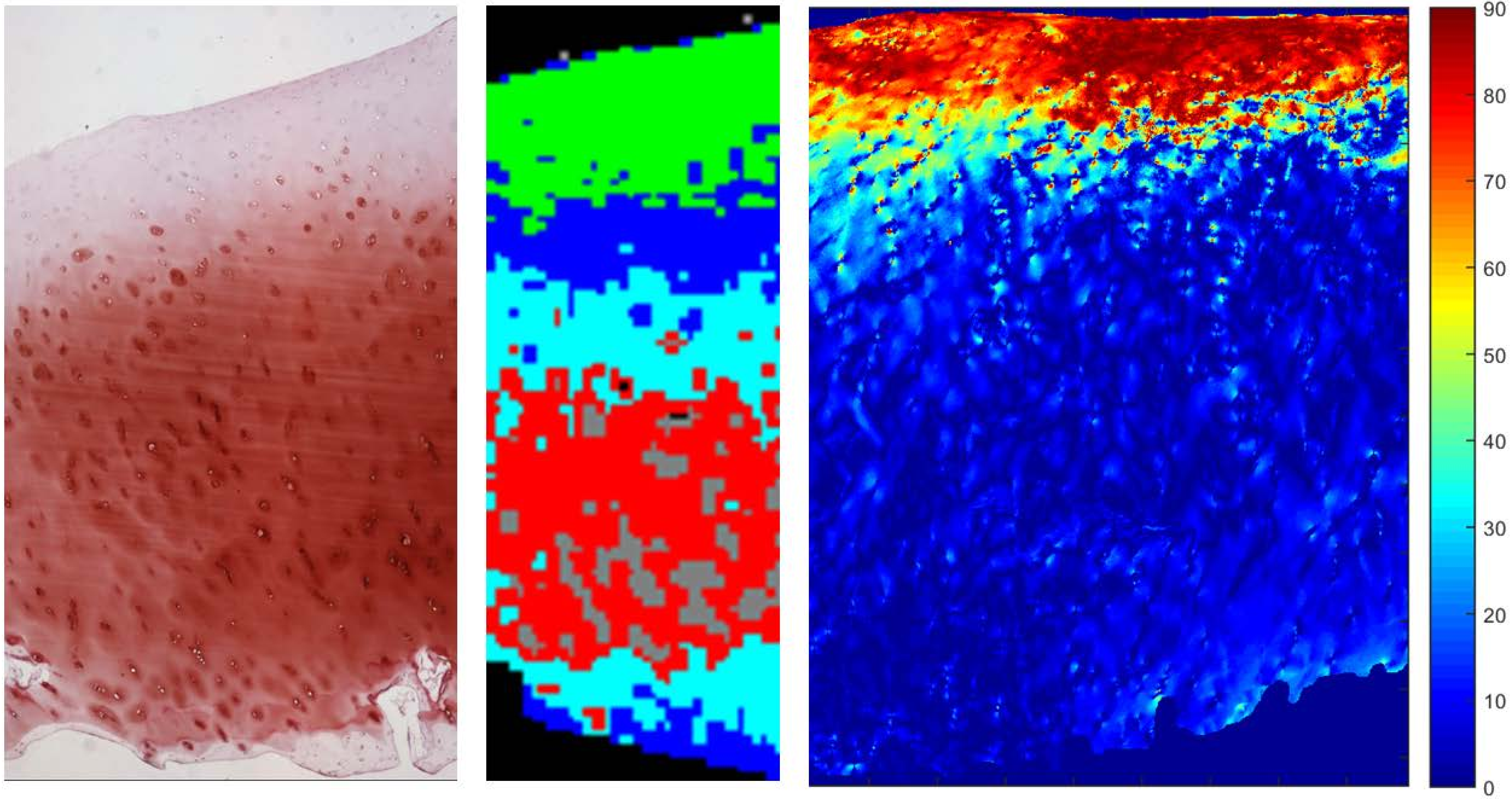

4. (OARSI grade 2.0)

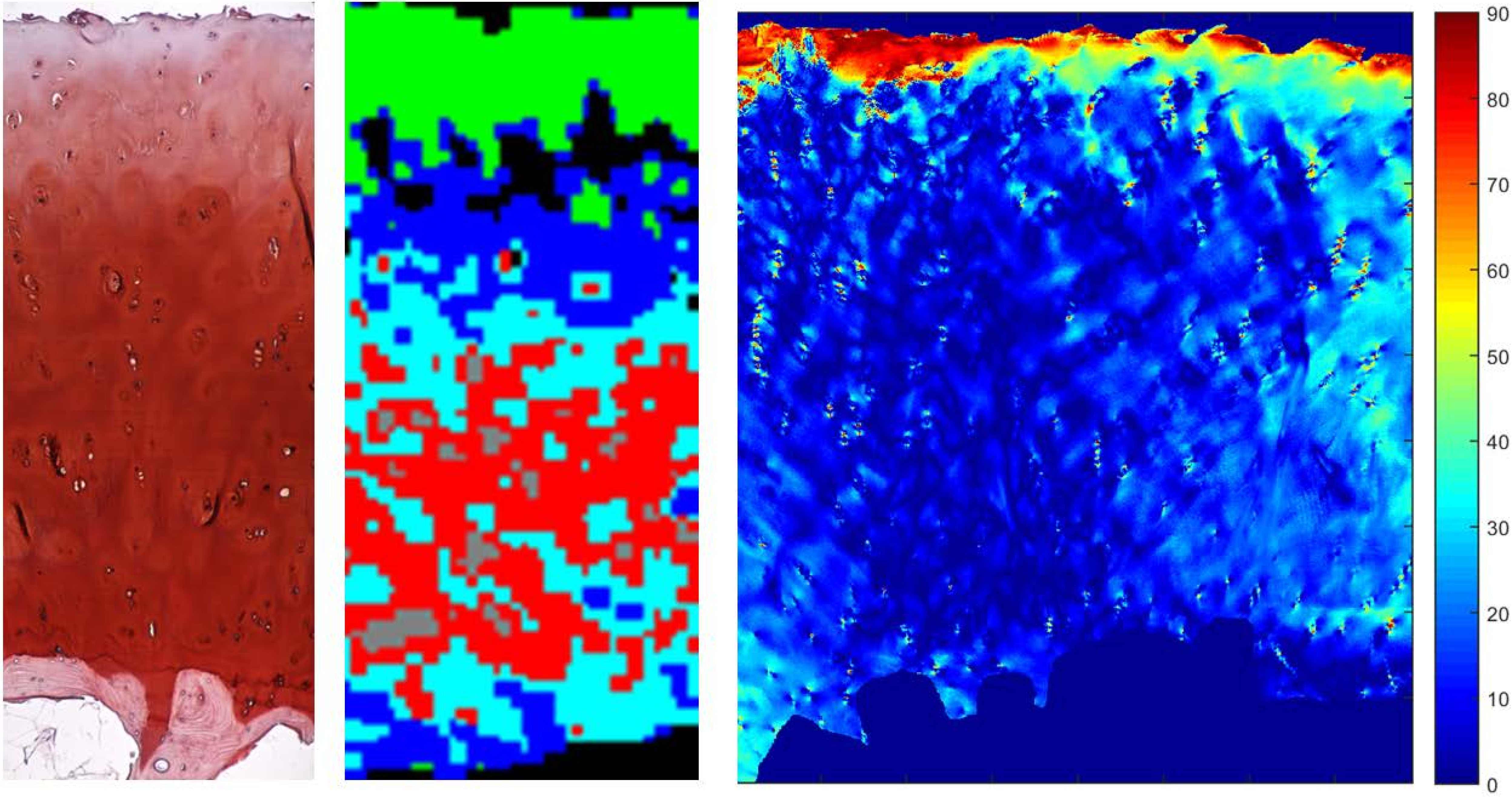

5. (OARSI grade 2.5)

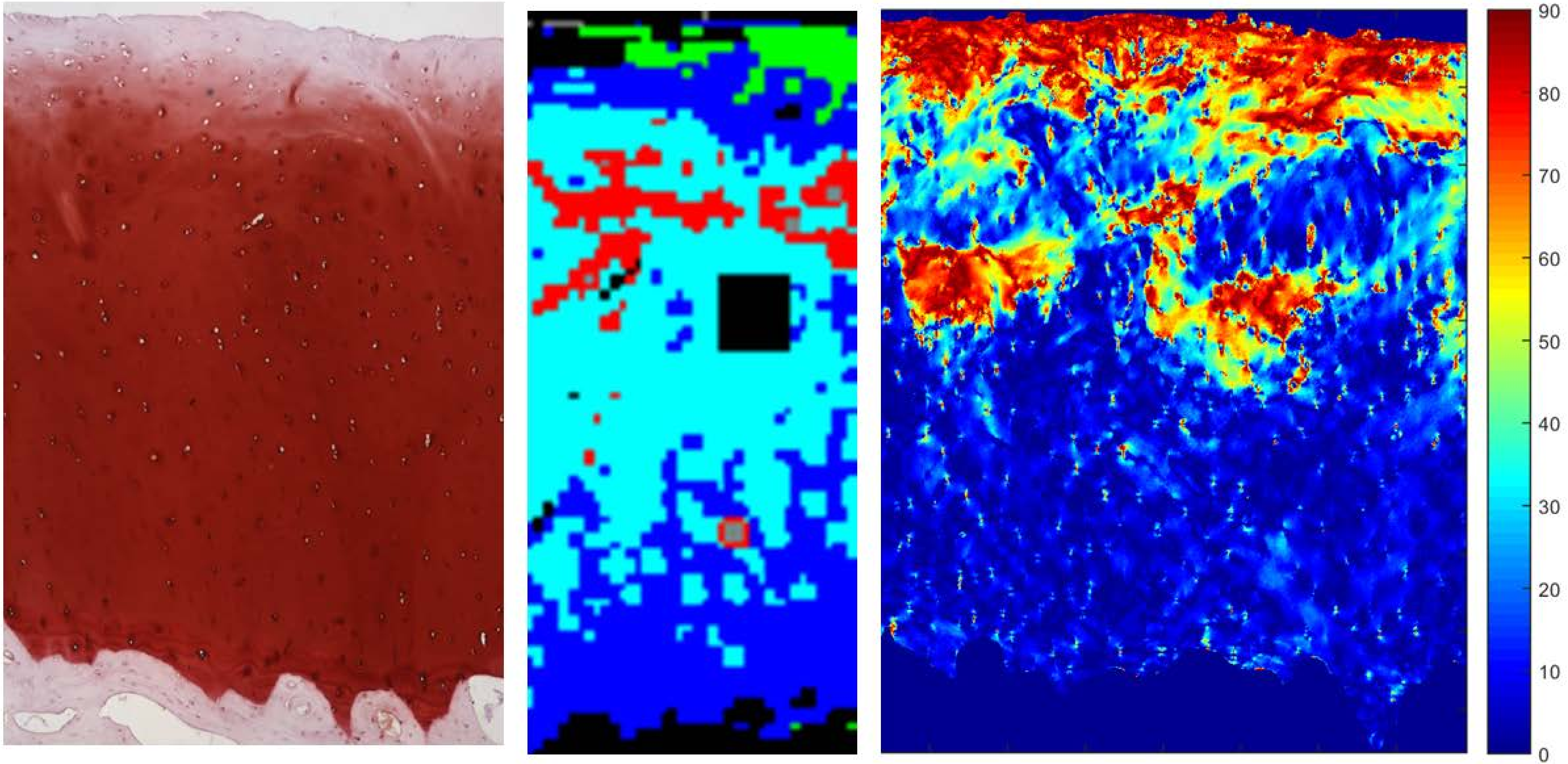

6. (OARSI grade 2.5)

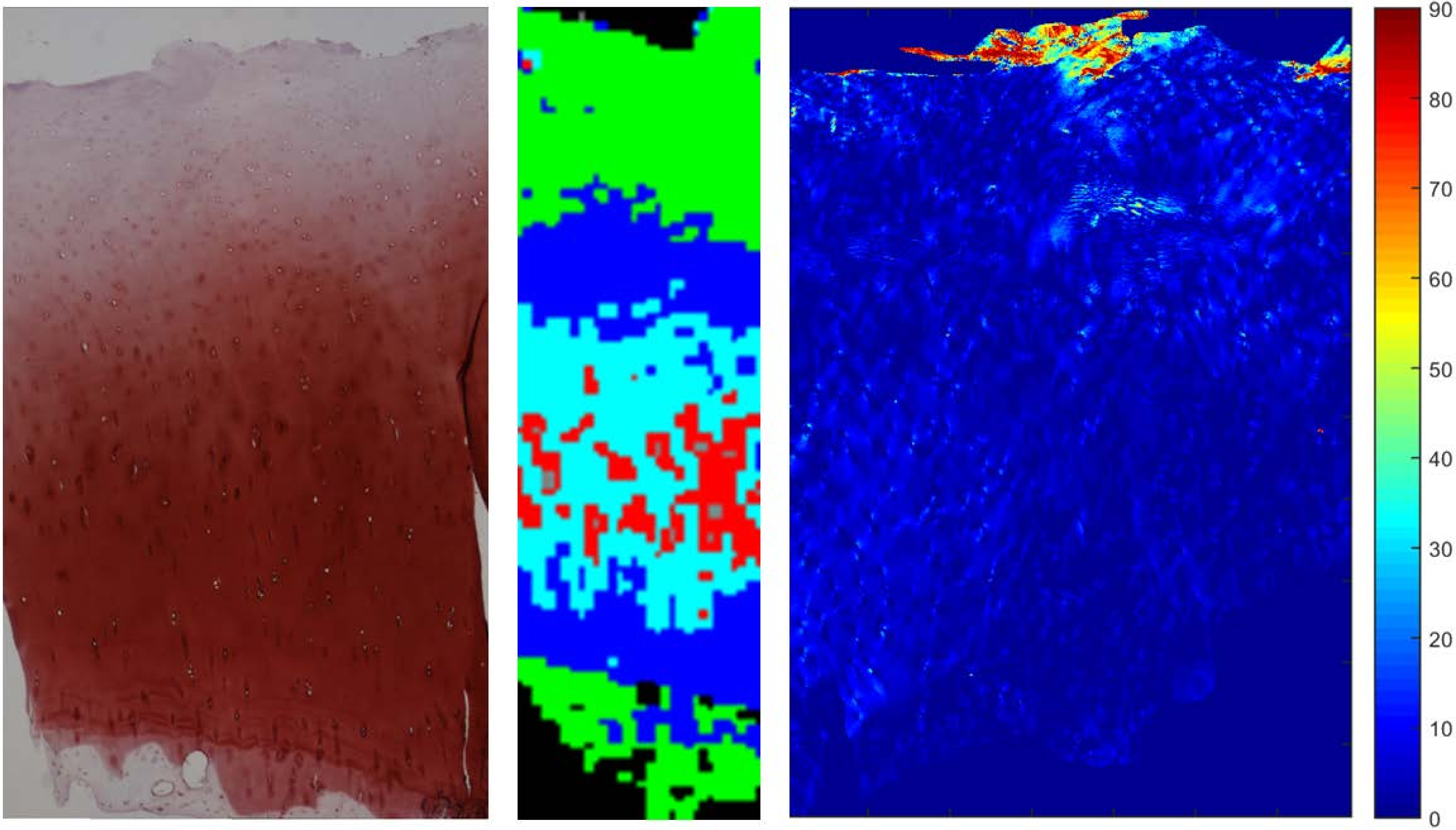

7. (OARSI grade 2.5)

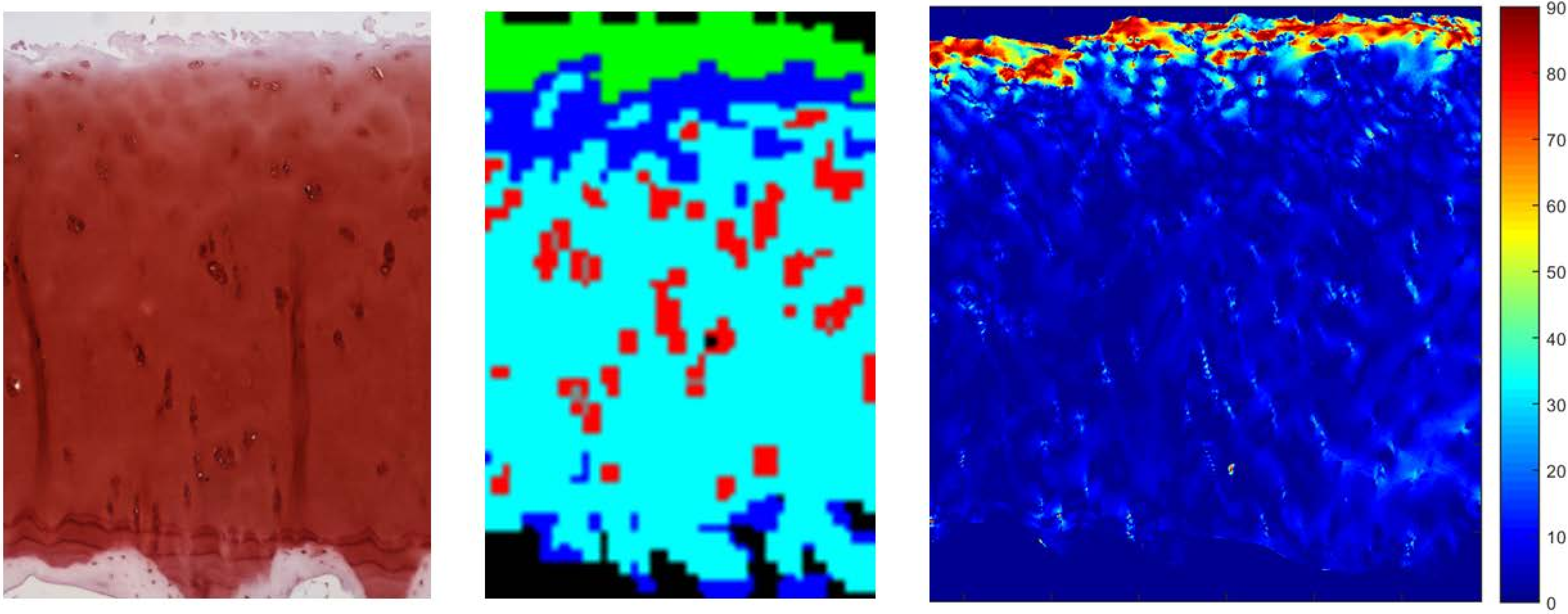

8. (OARSI grade 2.5)

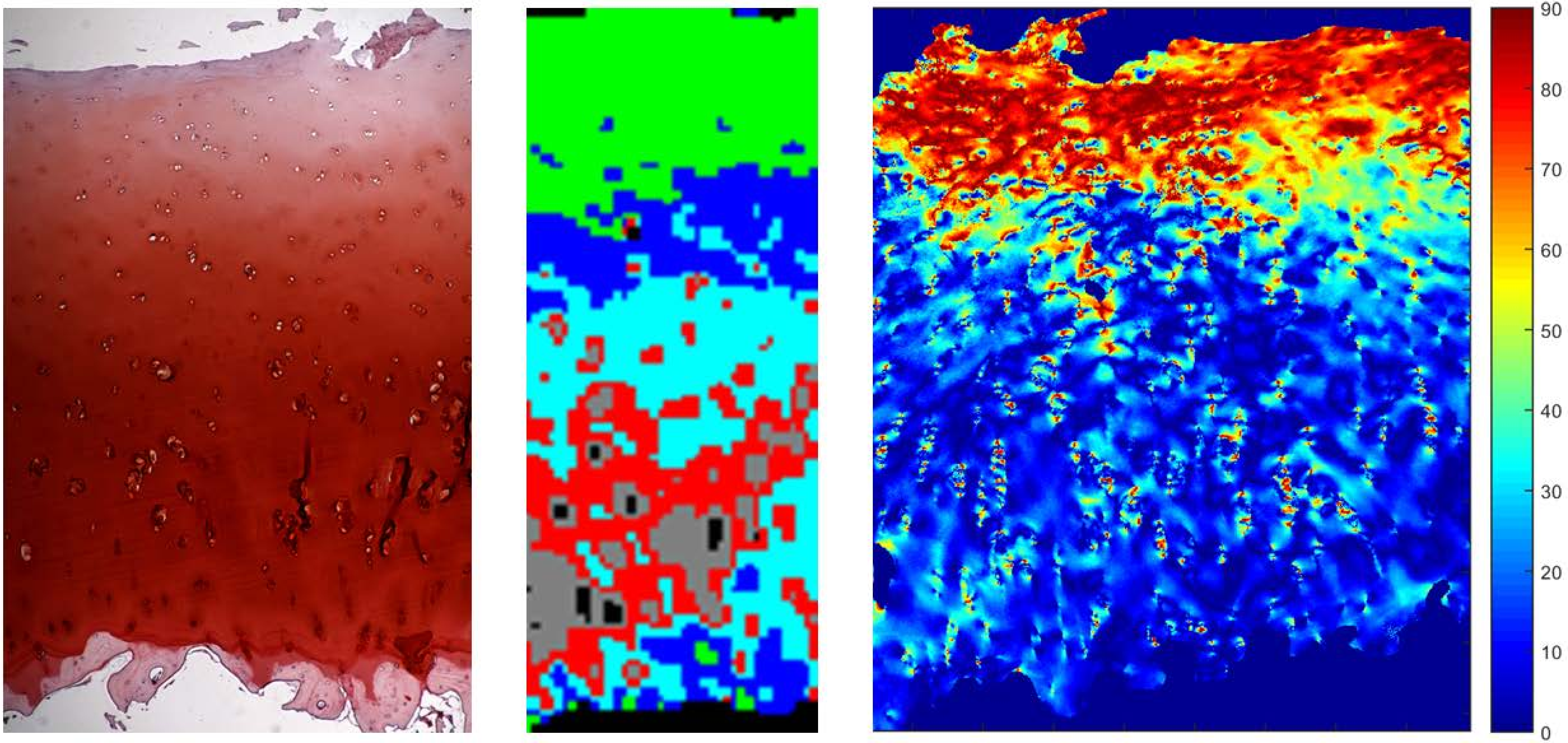

9. (OARSI grade 2.5)

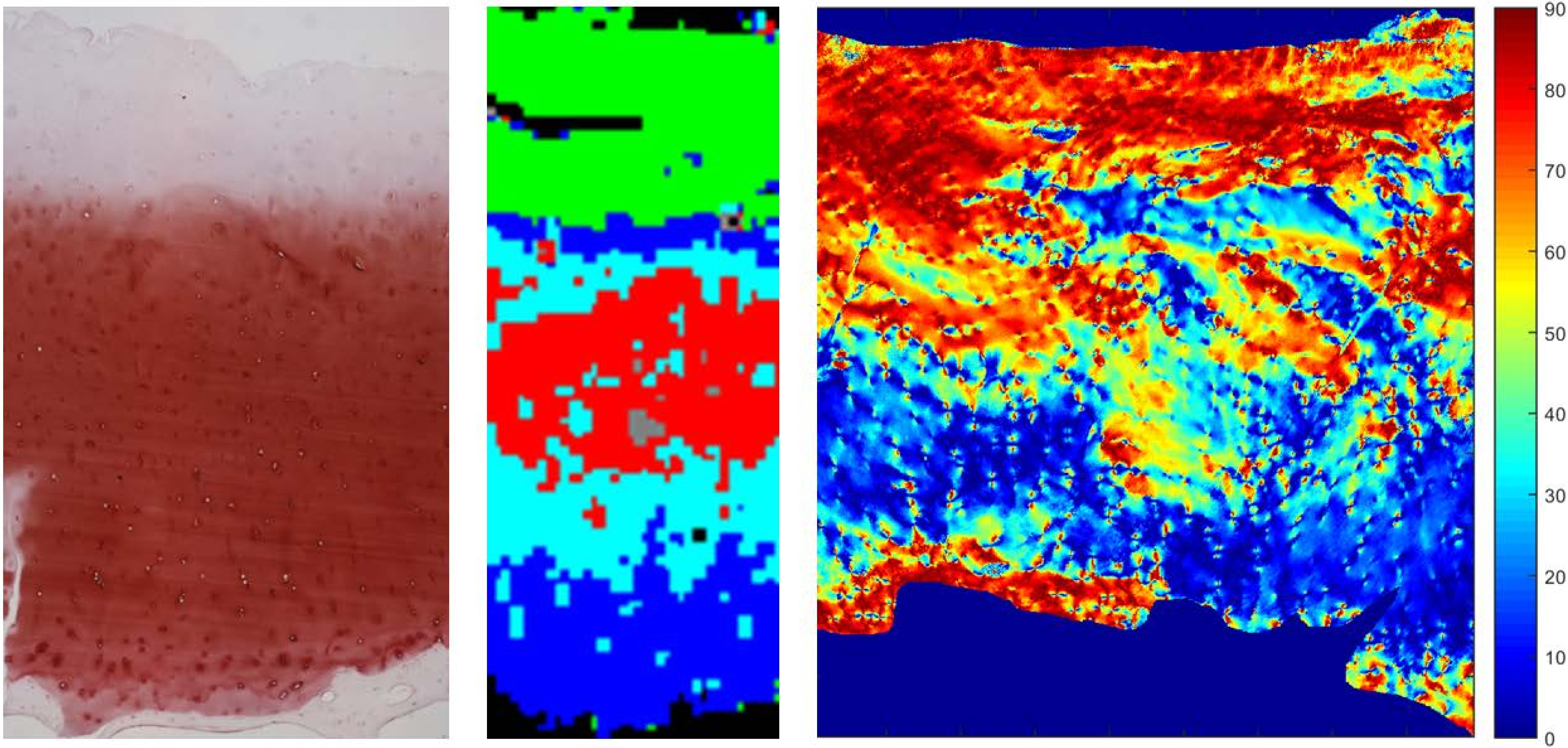

10. (OARSI grade 2.5)

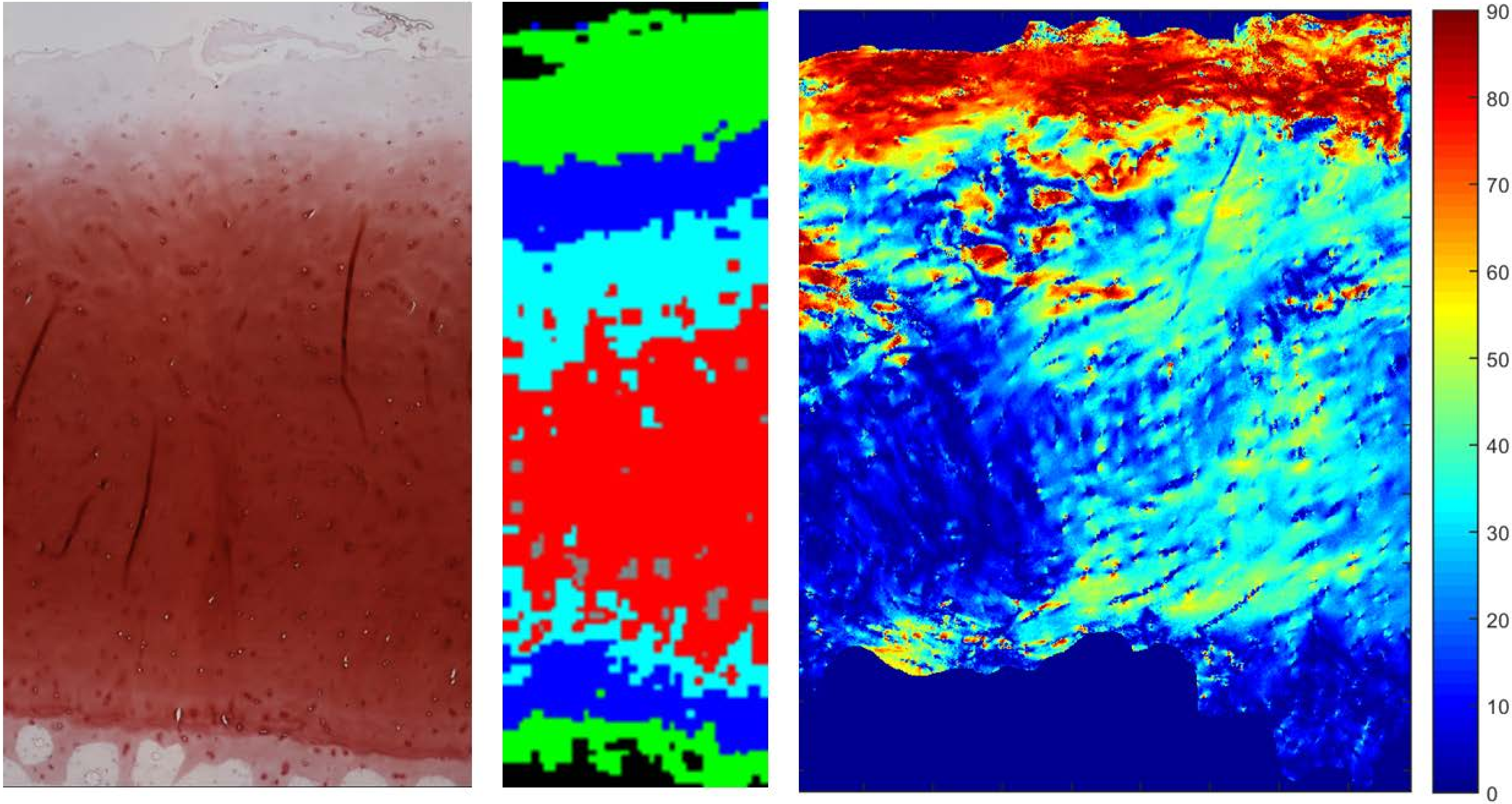

11. (OARSI grade 3.0)

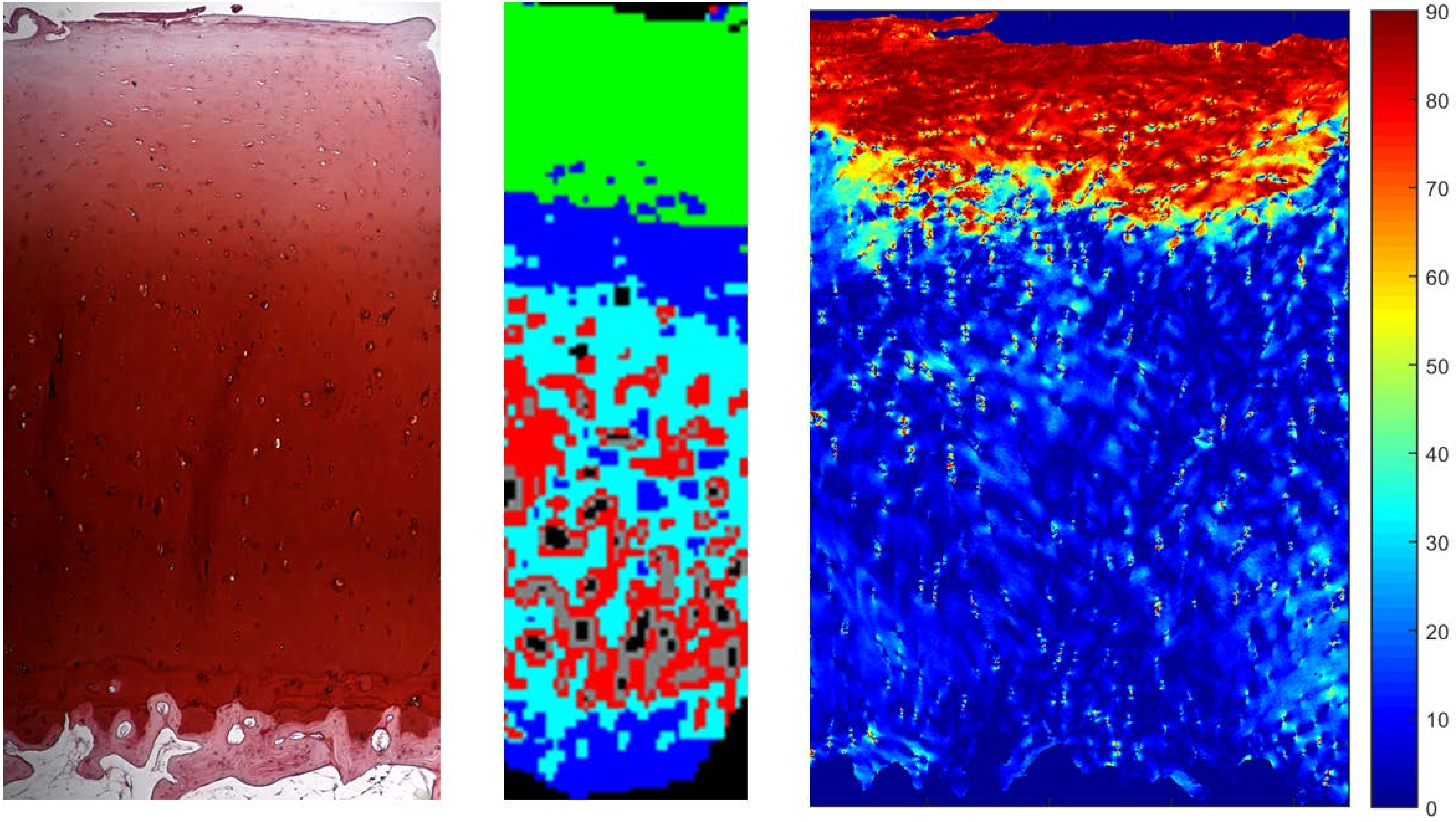

12. (OARSI grade 3.0)

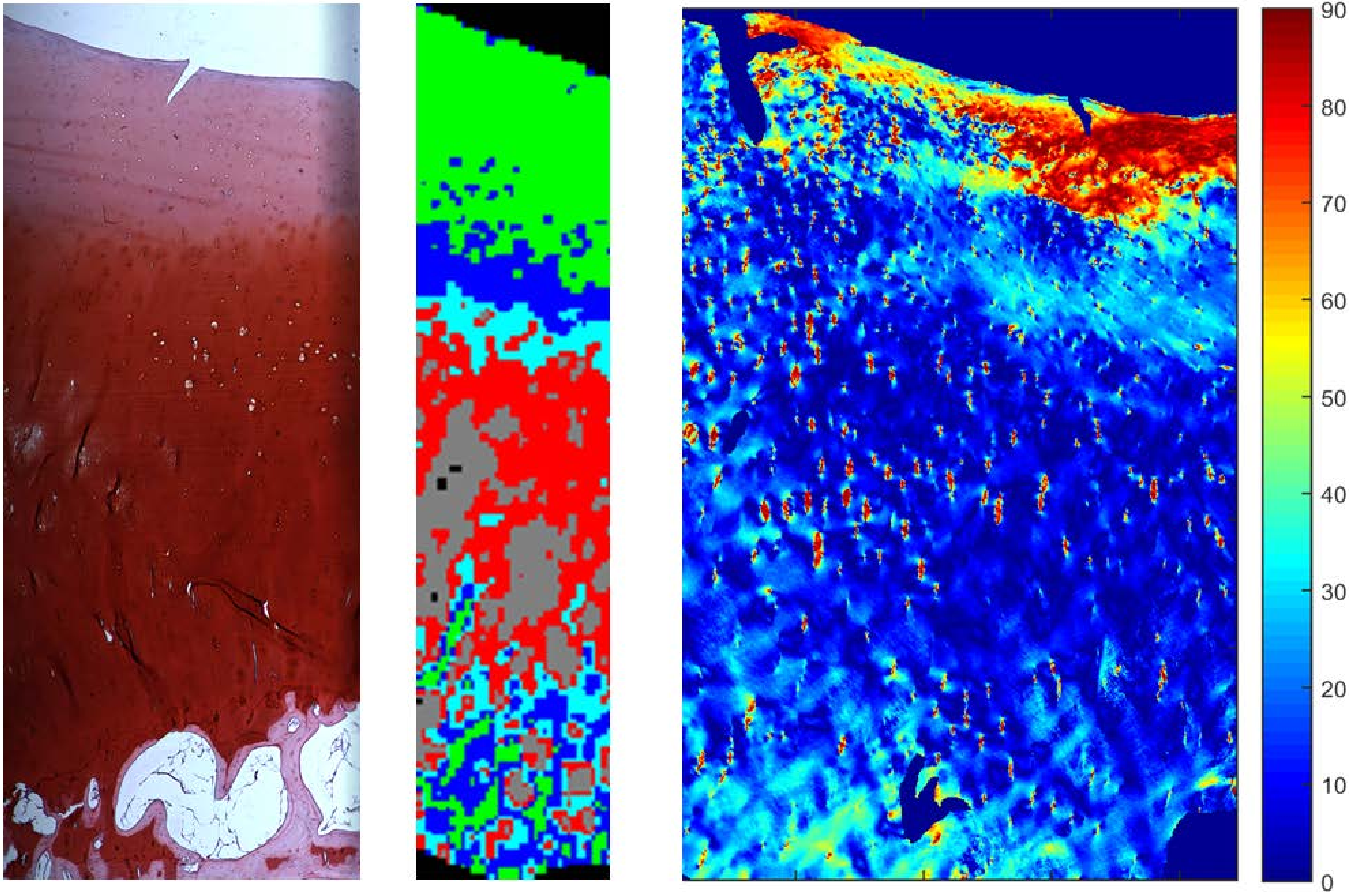

13. (OARSI grade 3.0)

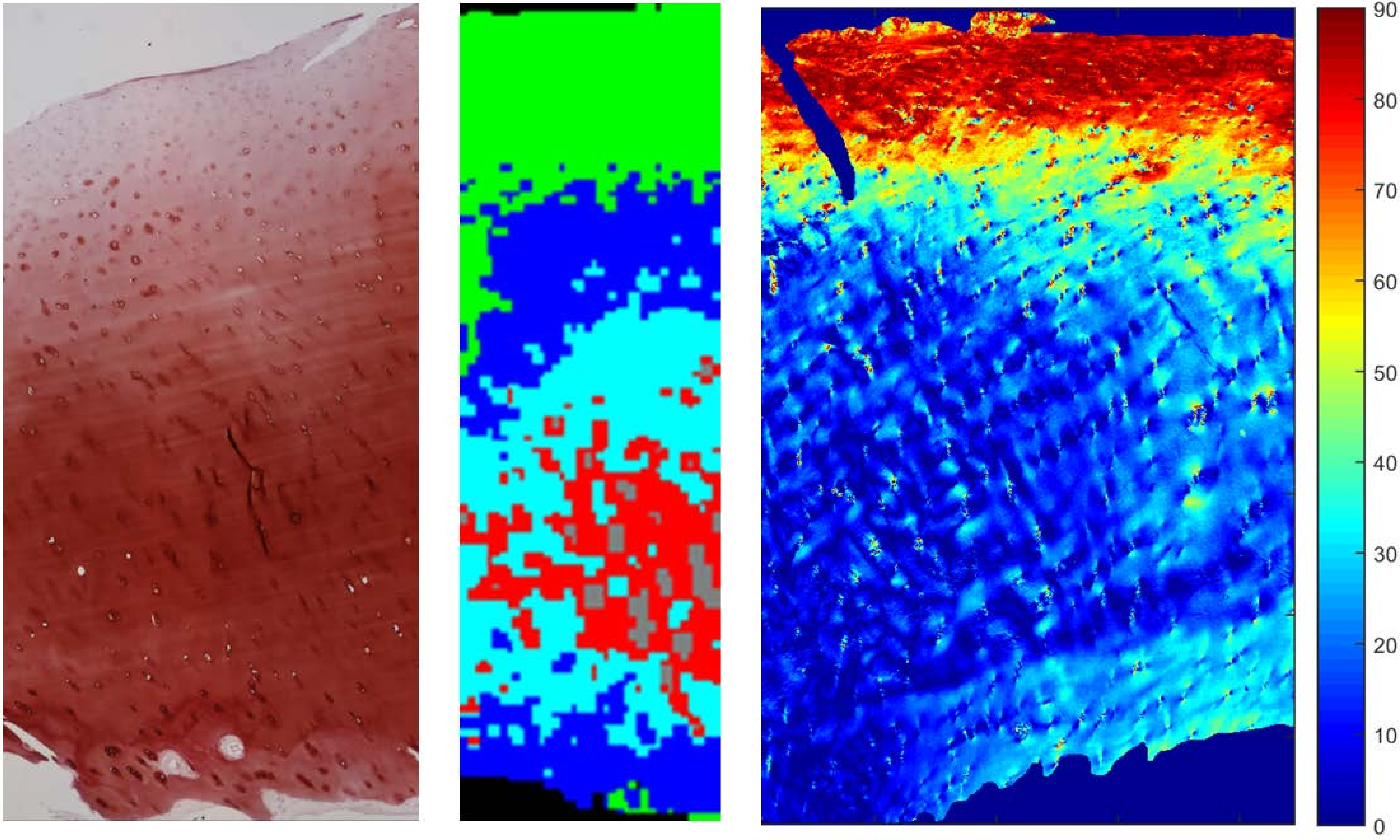

14. (OARSI grade 3.0)

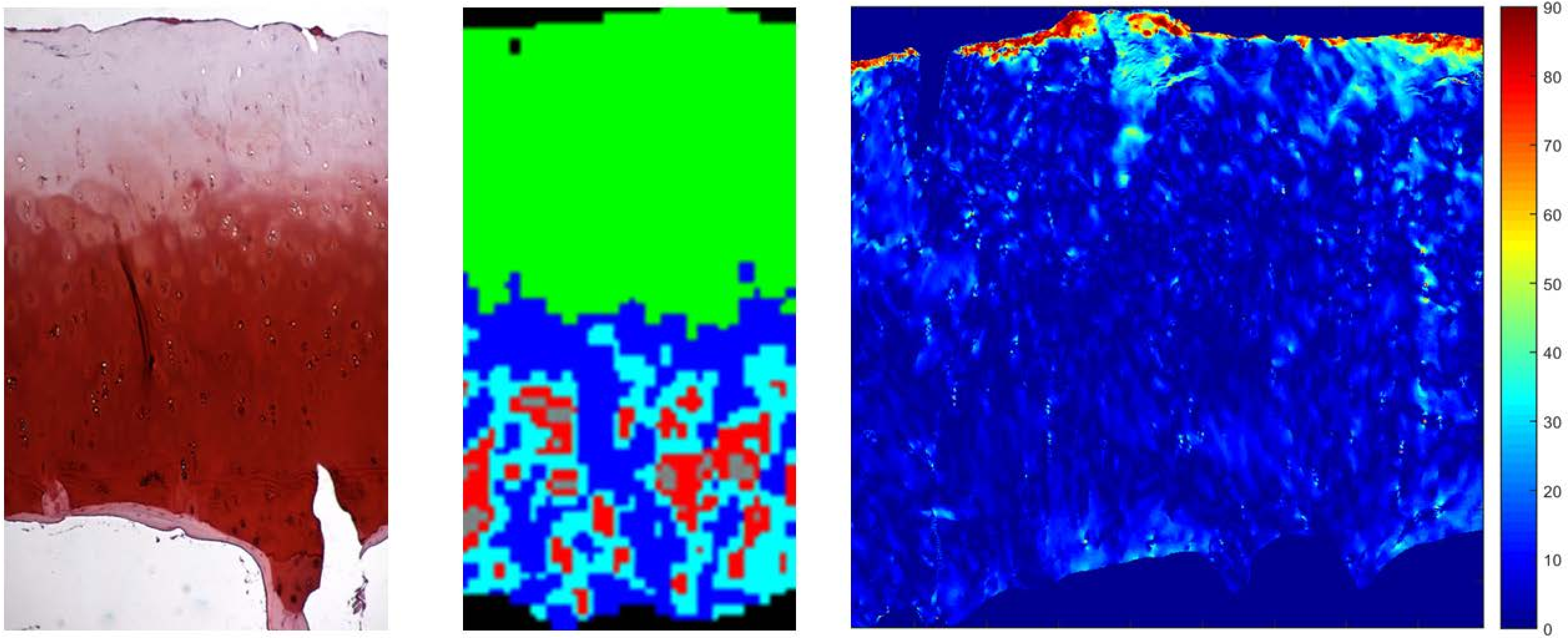

15. (OARSI grade 3.0)

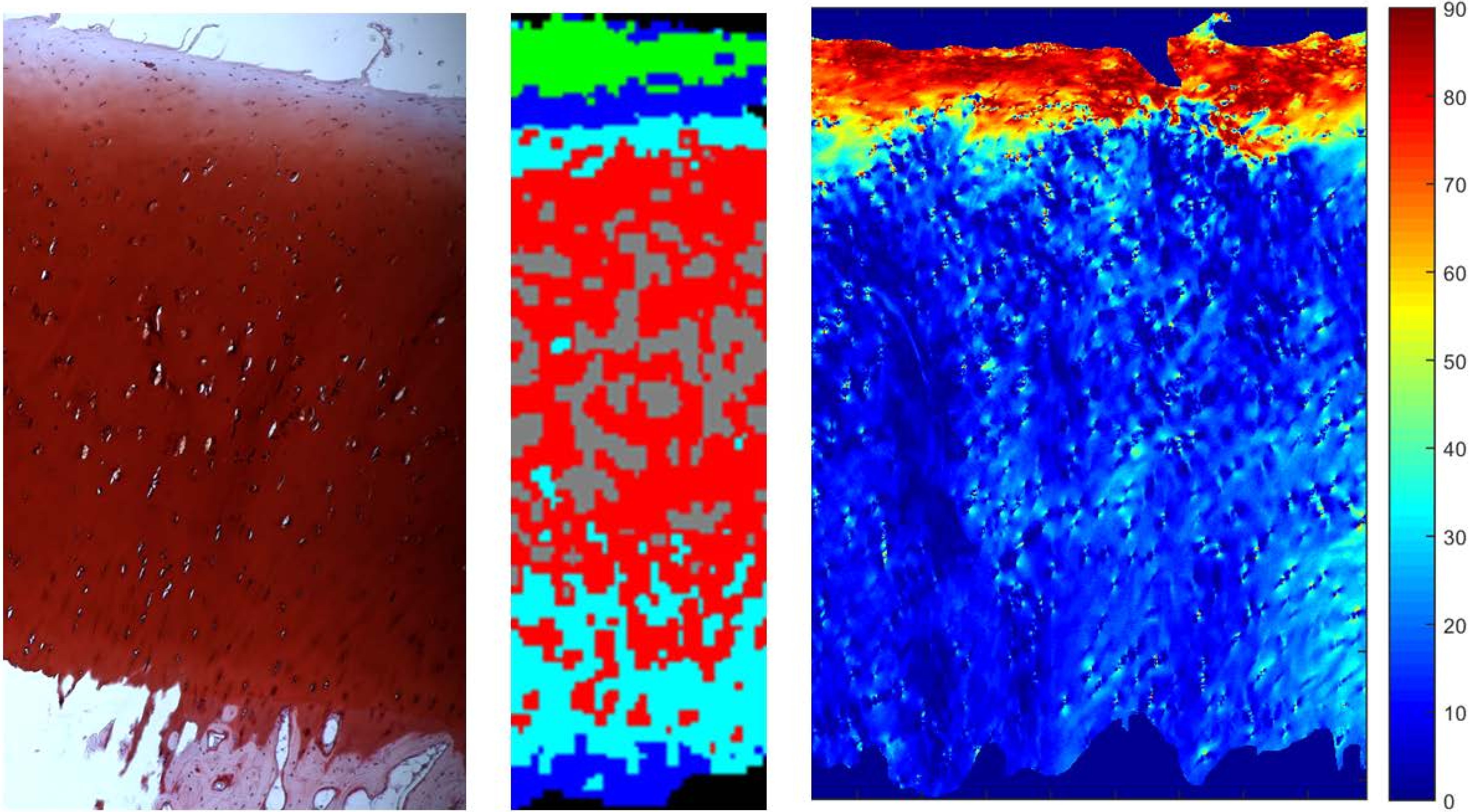

16. (OARSI grade 3.5)

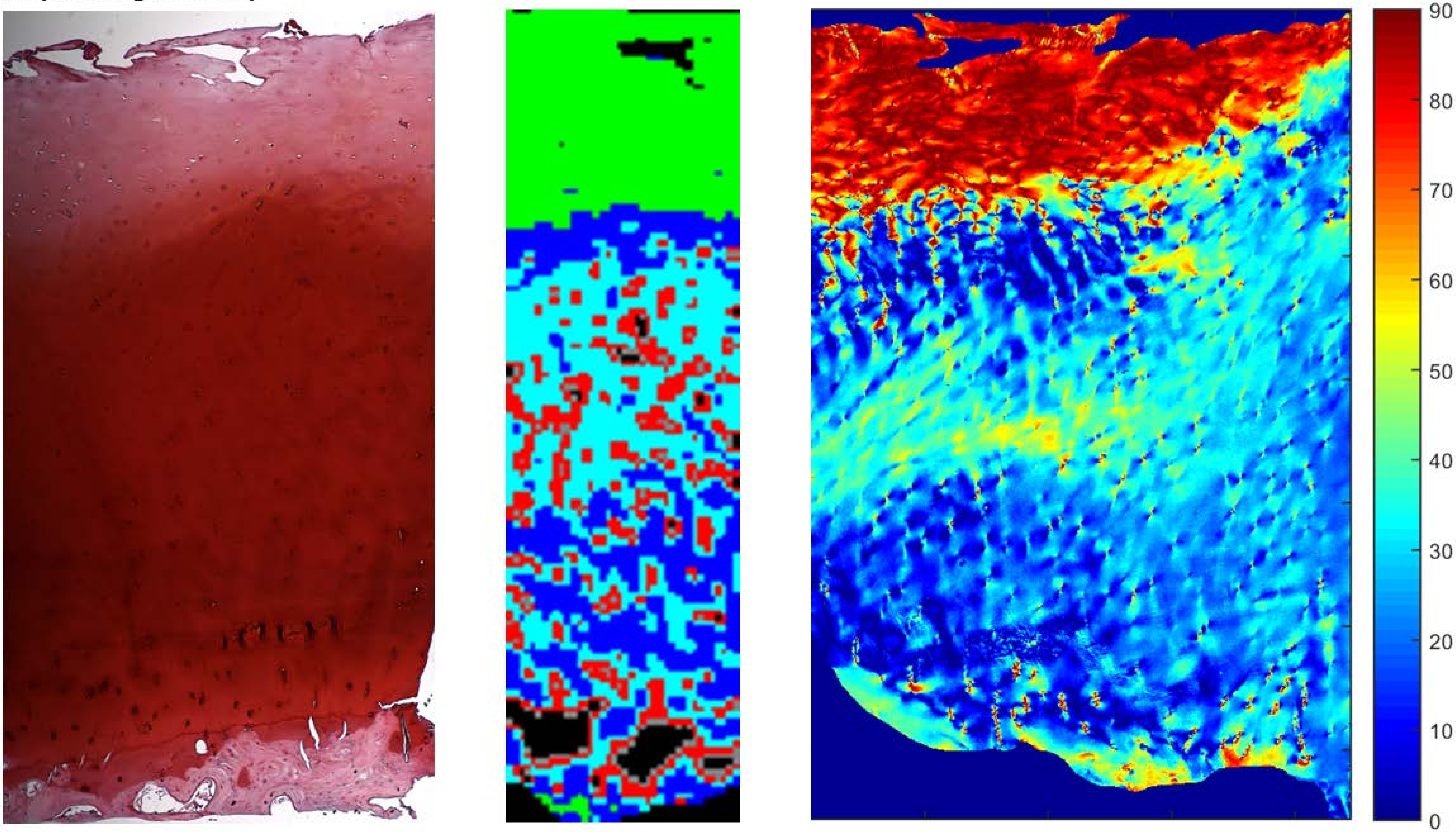

17. (OARSI grade 3.5)

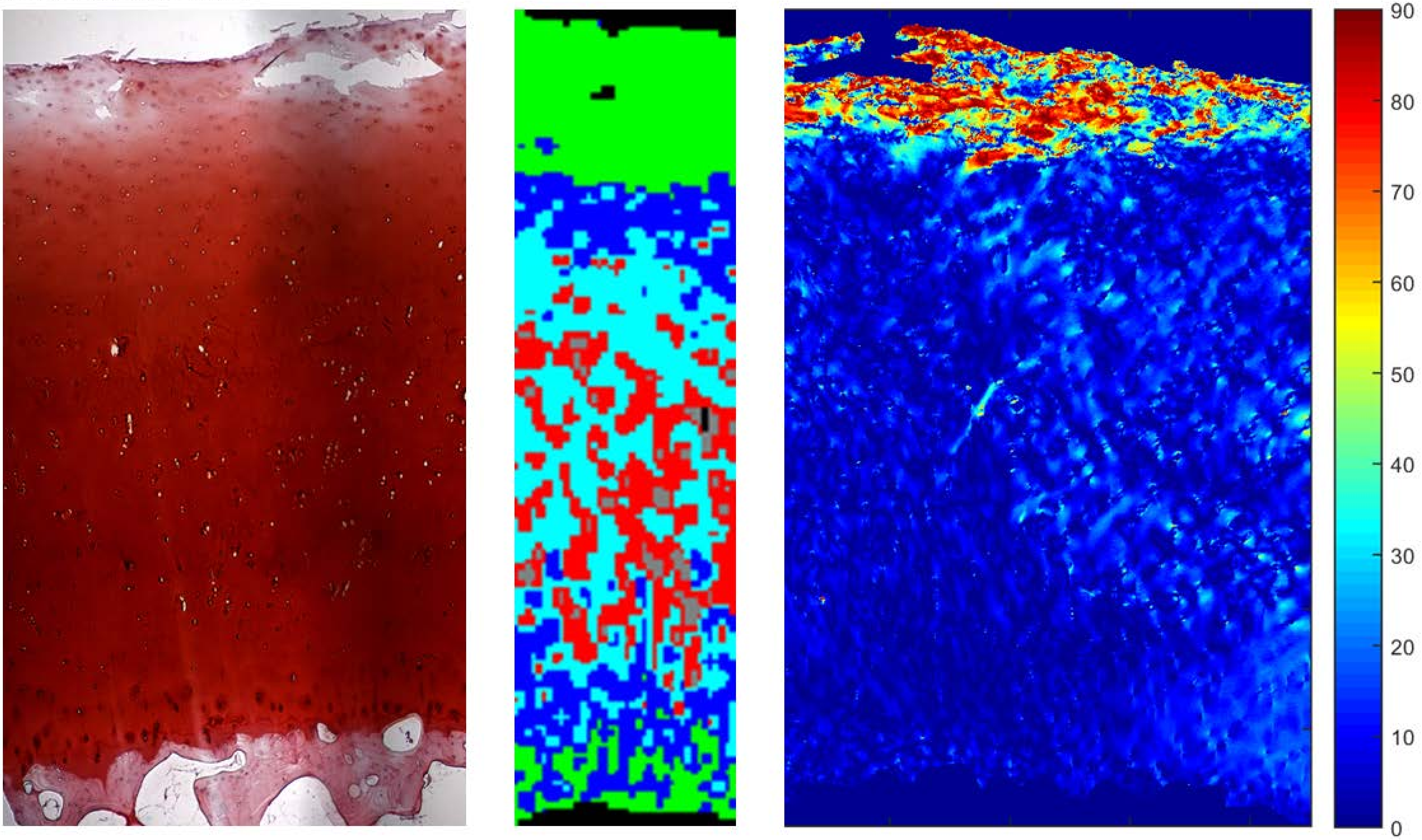

18. (OARSI grade 3.5)

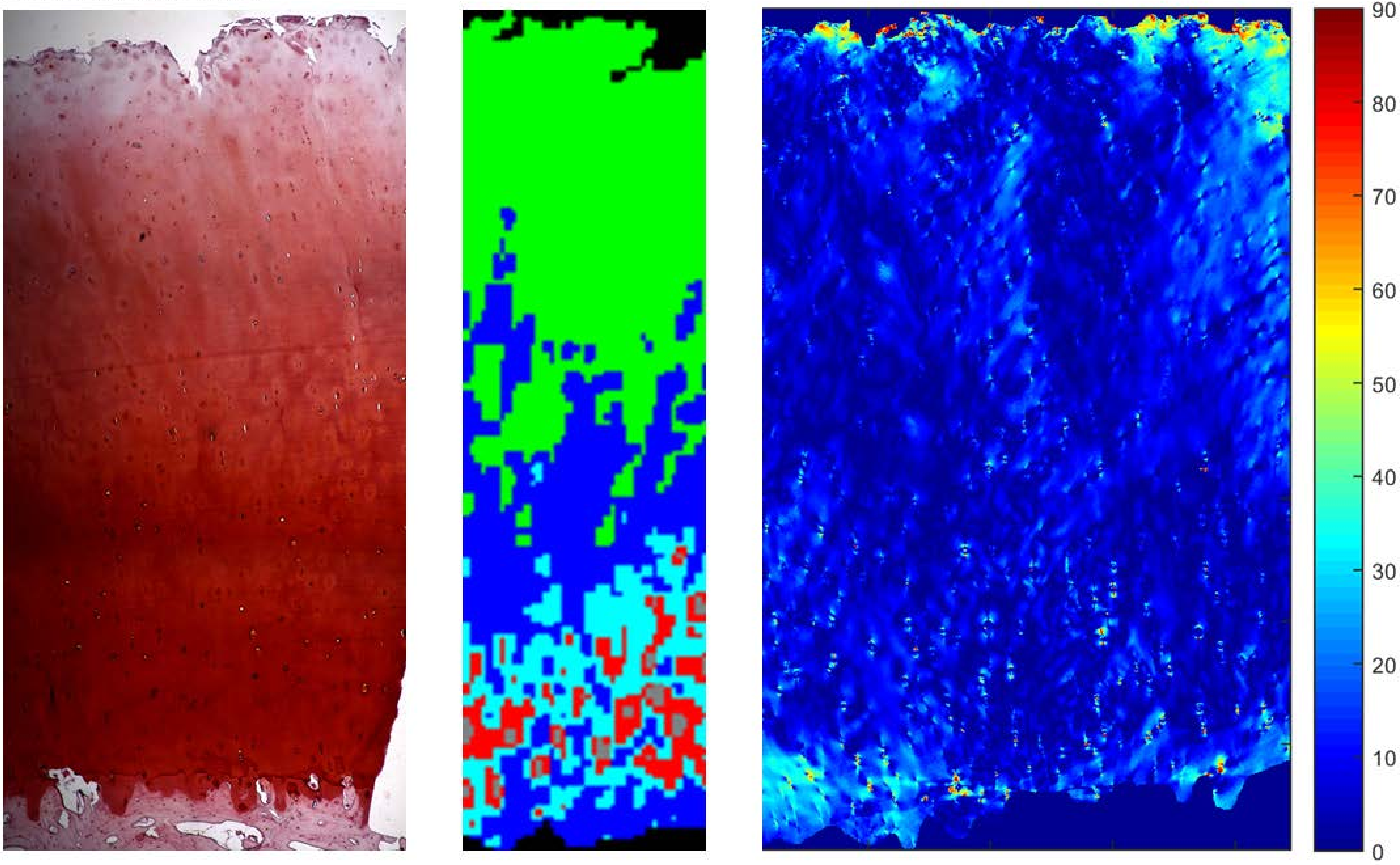

19. (OARSI grade 3.5)

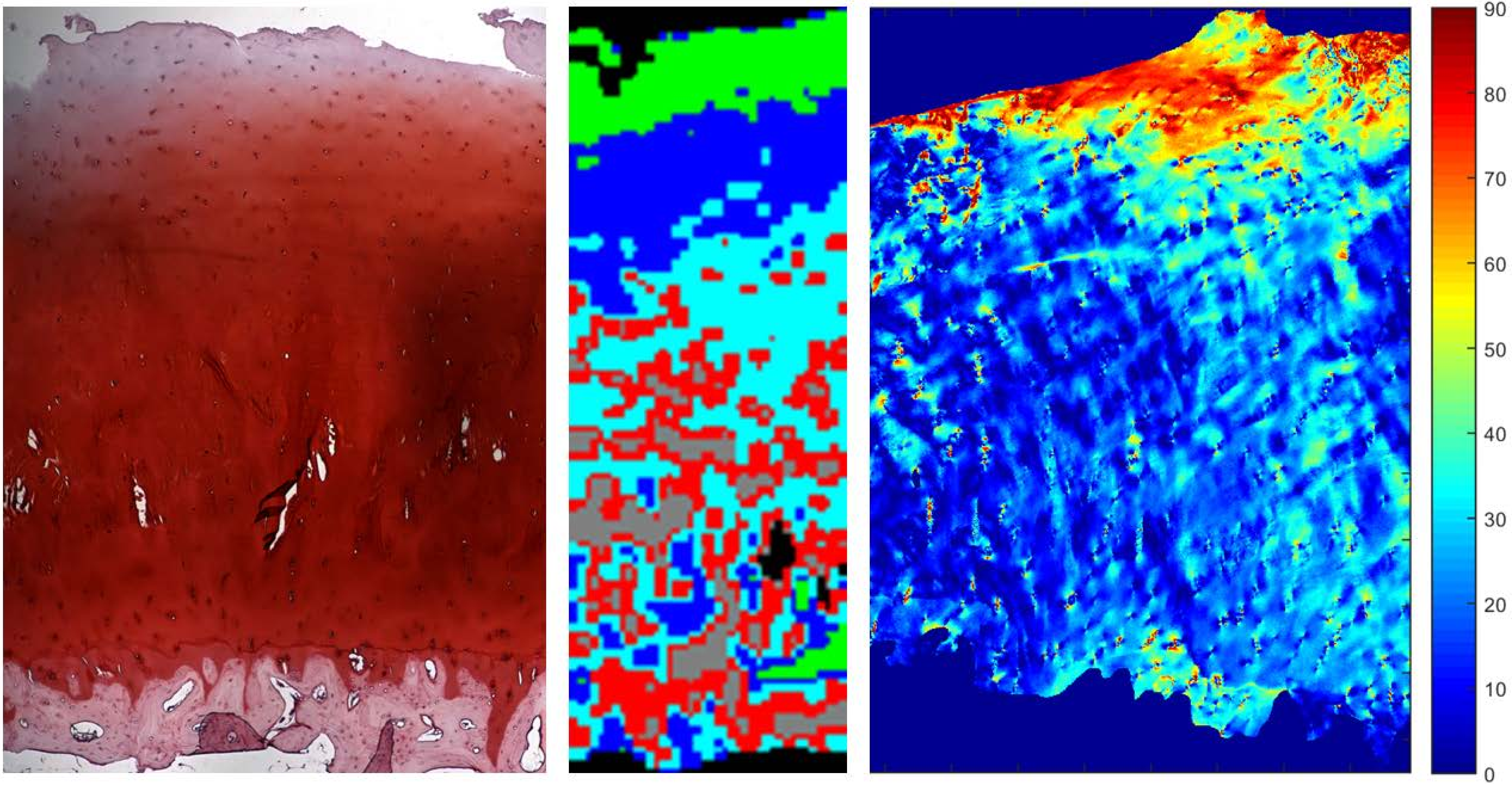

20. (OARSI grade 4.0)

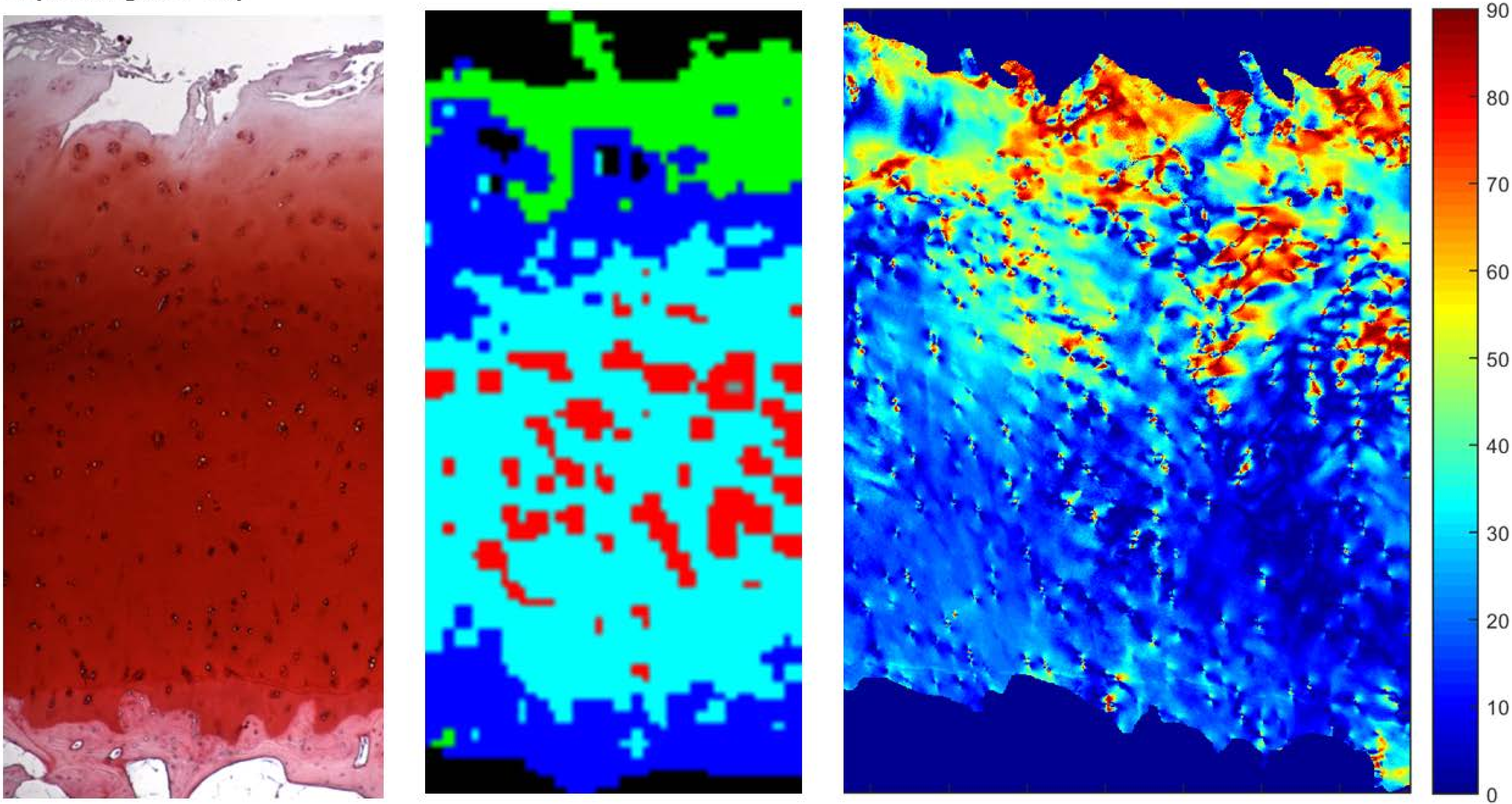

21. (OARSI grade 4.0)

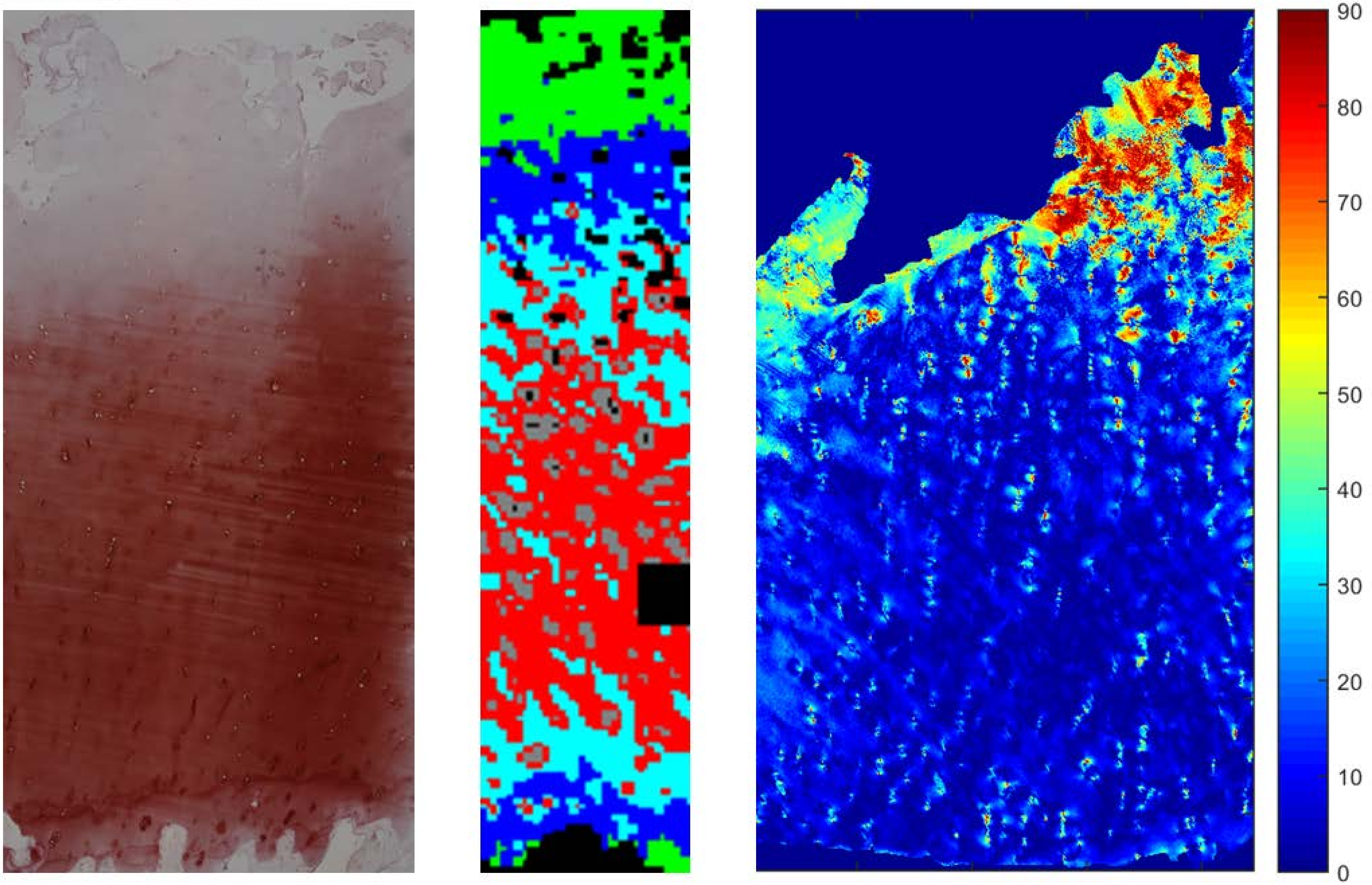

22. (OARSI grade 4.0)

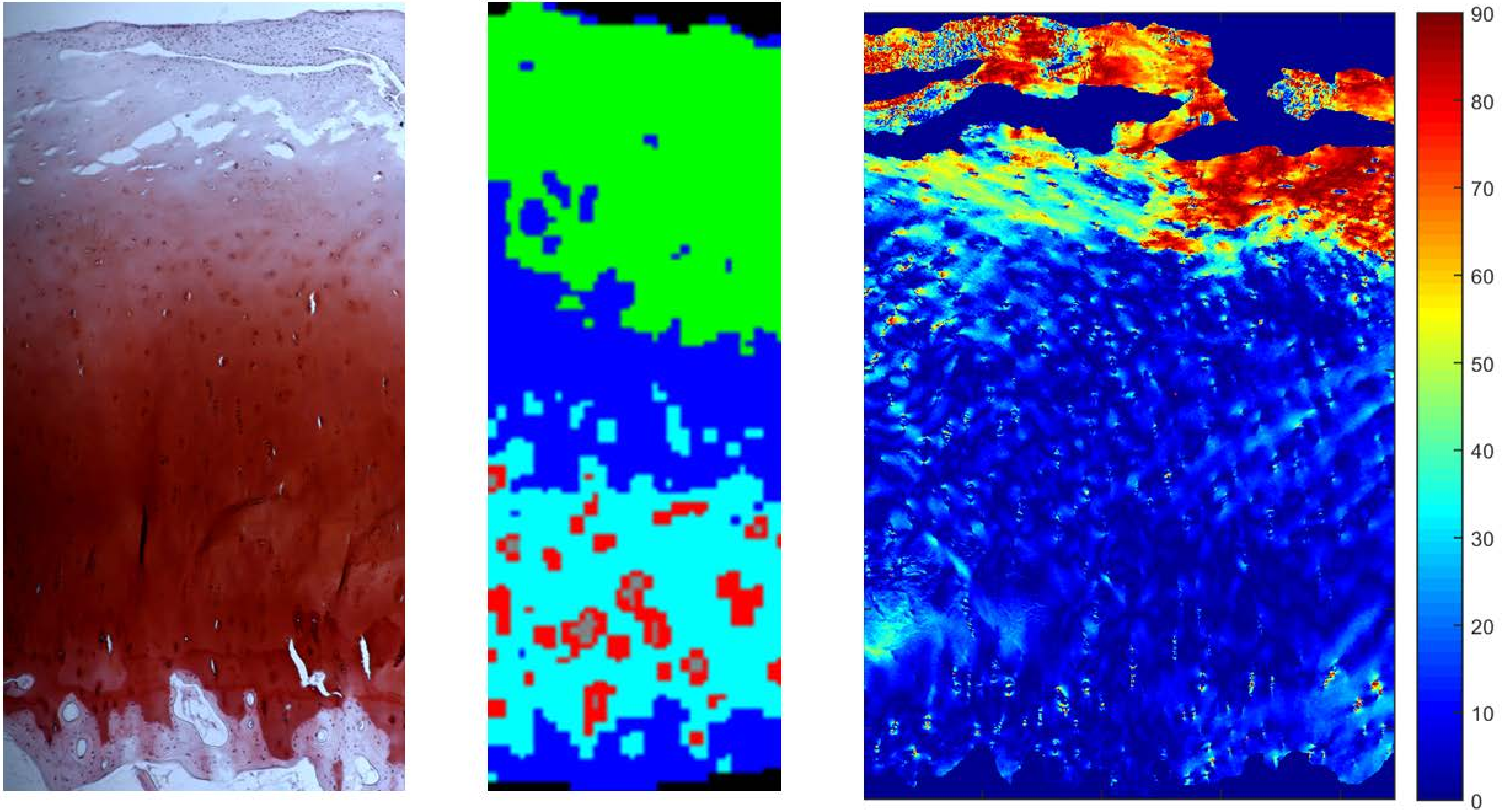

23. (OARSI grade 4.5)

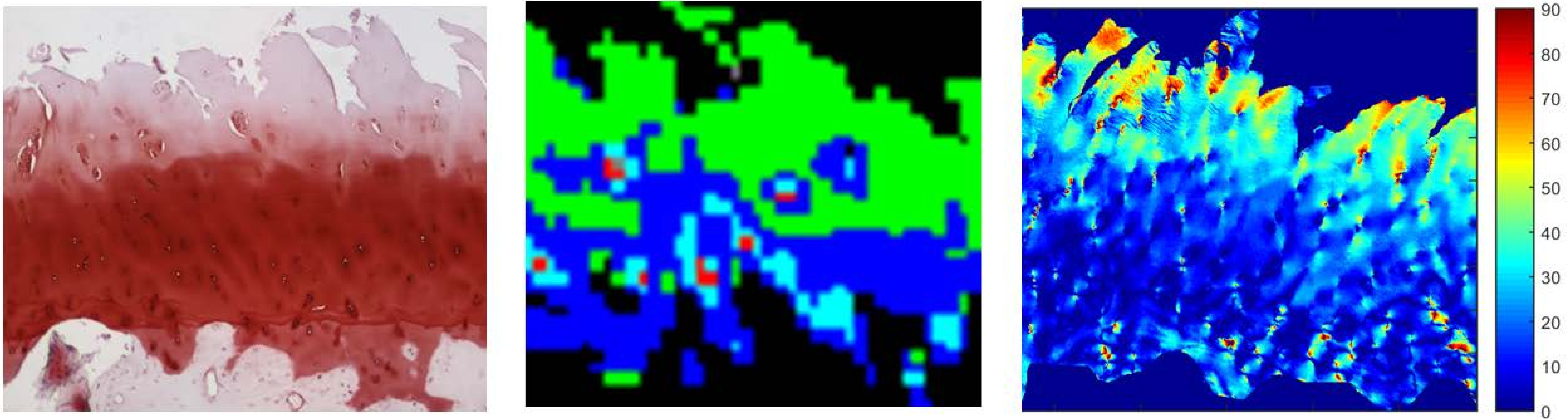

24. (OARSI grade 4.5)

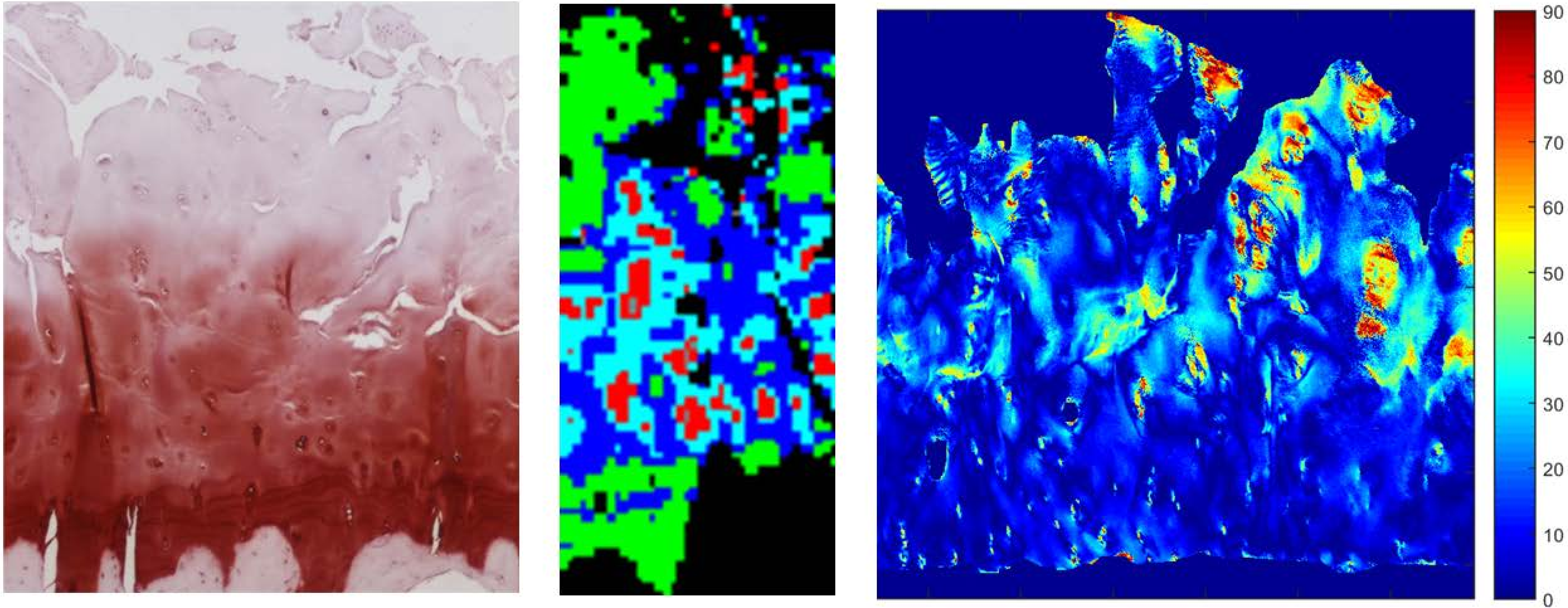

25. (OARSI grade 4.5)

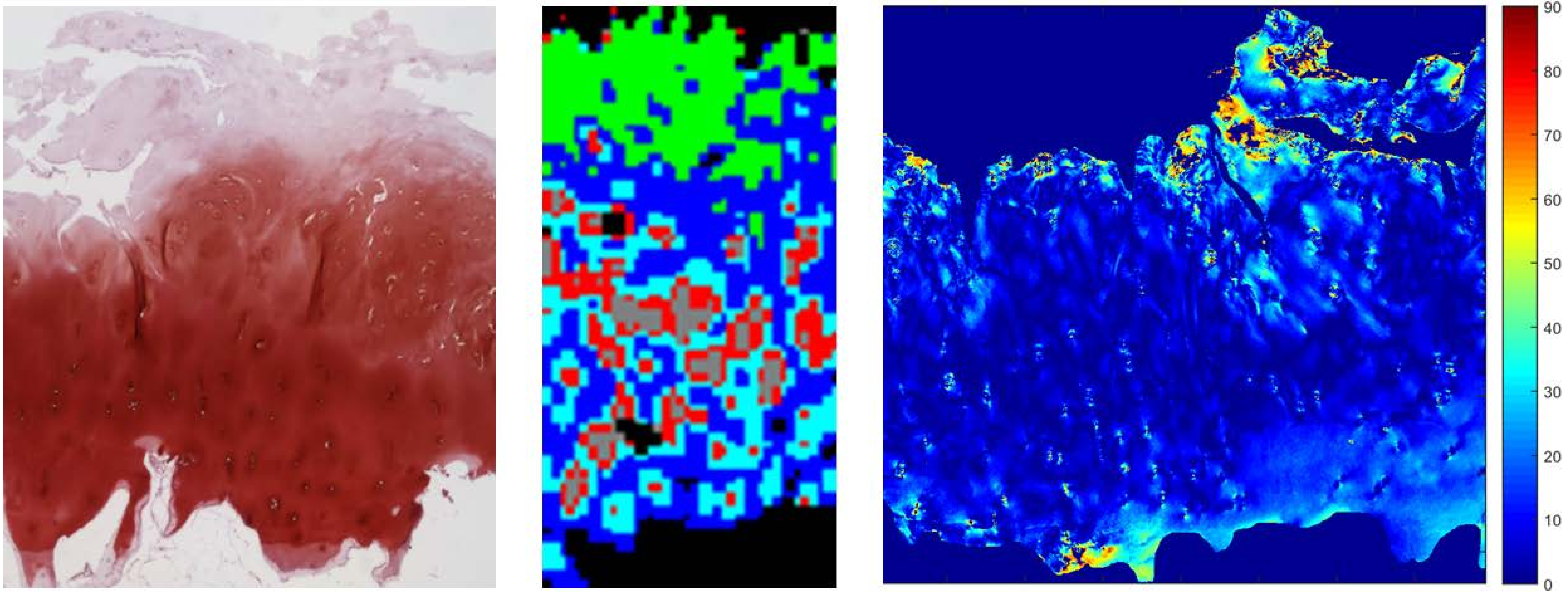

26. (OARSI grade 4.5)

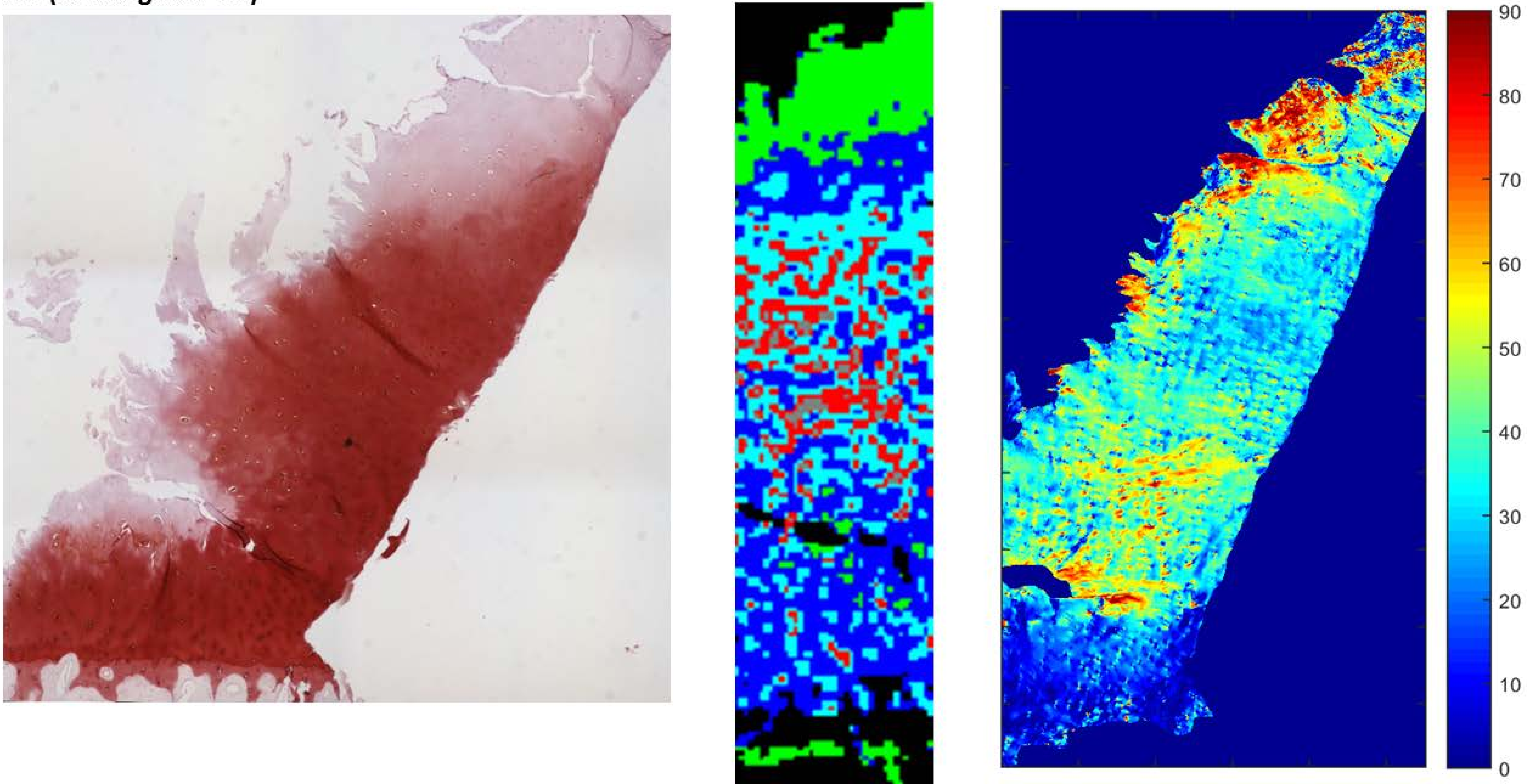

27. (OARSI grade 4.5)

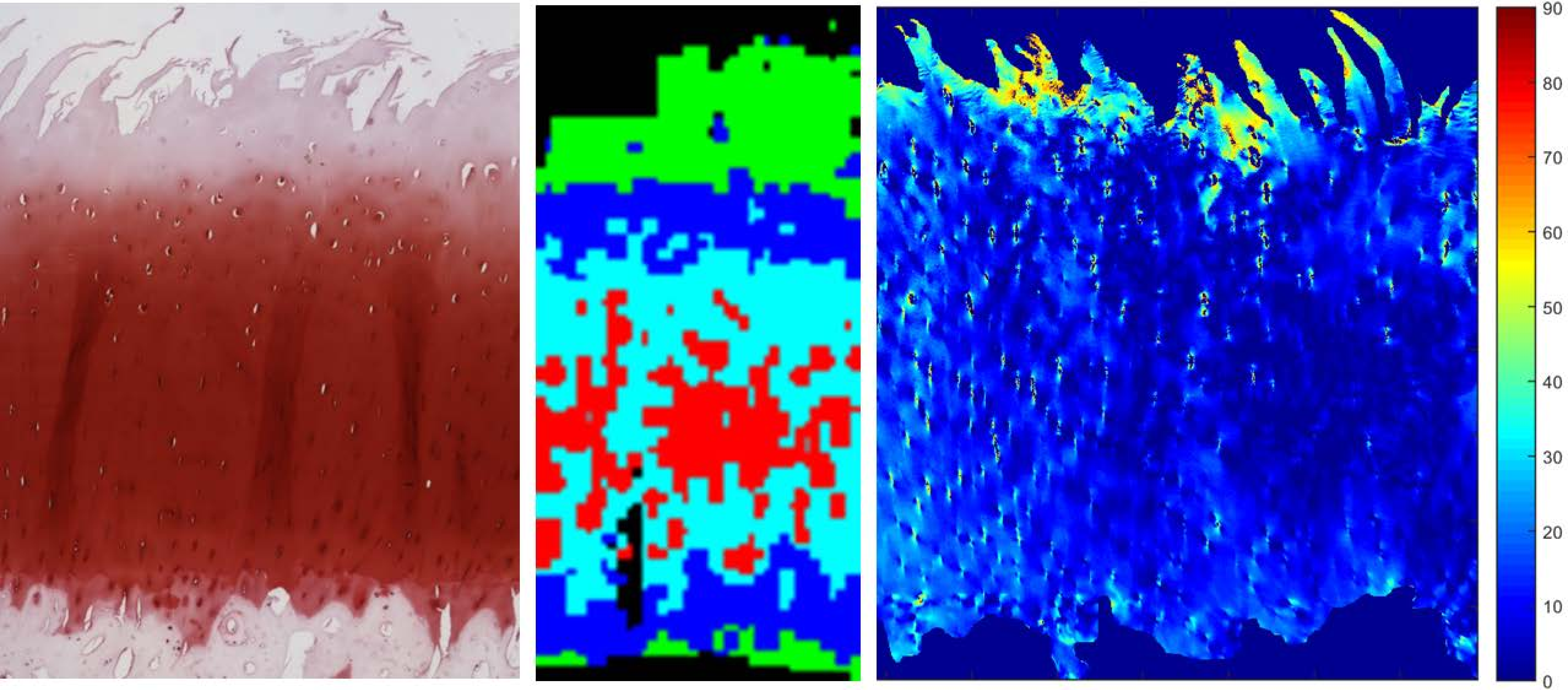

28. (OARSI grade 4.5)

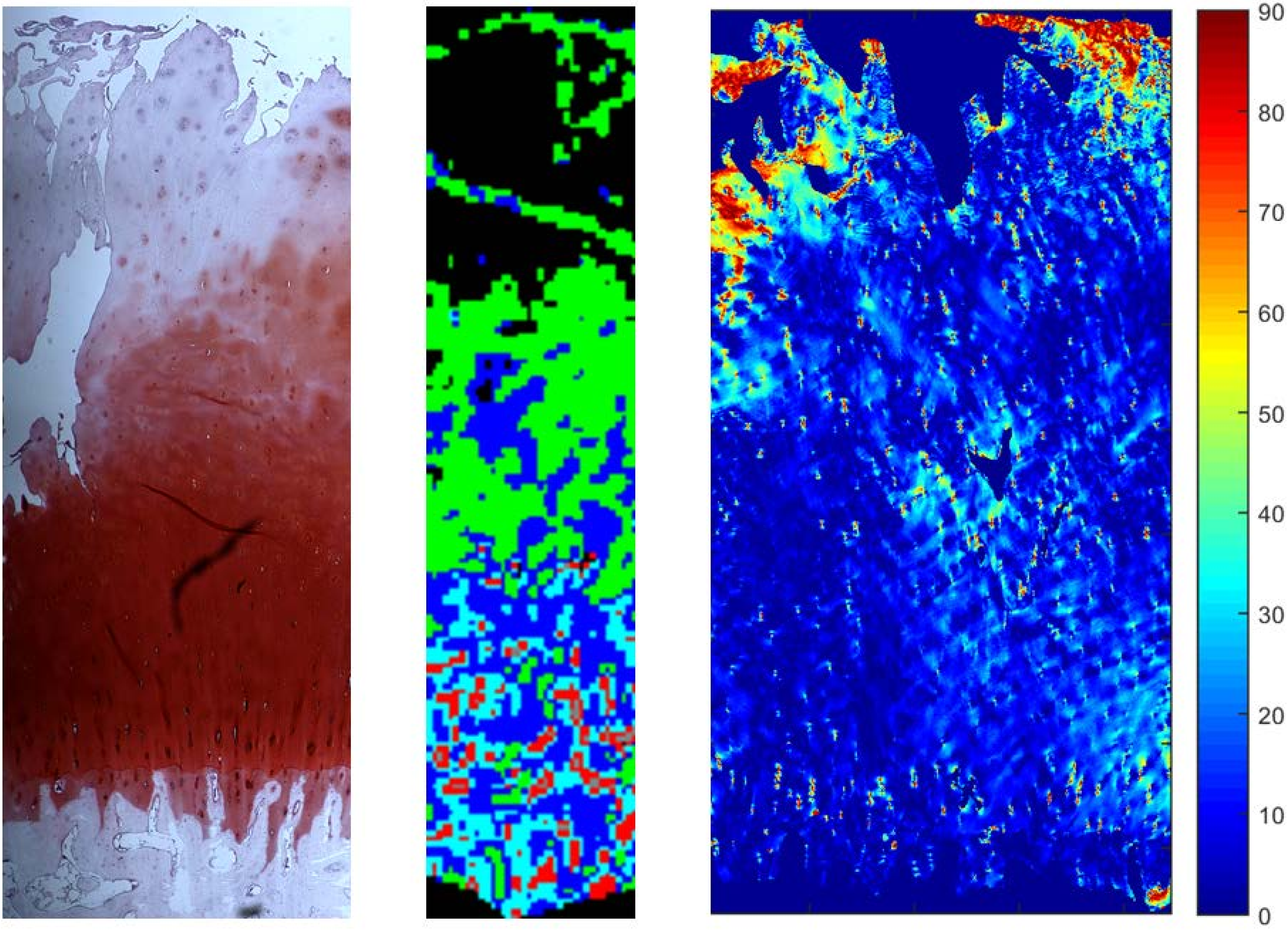

Supplement: Supplementary Information [file srep30008-s1.pdf]
